# Ubiquitin-specific protease 7-mediated stabilization of discoidin domain receptor 1 drives progression of TP53-Mutant cancers

**DOI:** 10.1016/j.jbc.2025.110515

**Published:** 2025-07-24

**Authors:** Yiying Xue, Bing Xiu, Muye Yang, Yanwei Zhang, Changpeng Yang, Lele Zhang, Yilv Yan, Xiu Luo, Yushan Huang, Husheng Mei, Guiming Li, Lixin Zhou, Yisa Chen, Wenjun Zhang, Changlin Qian, Peng Zhang, Juan Liu, Aibin Liang, Yu Zeng, Jing Yang

**Affiliations:** 1 Department of Hematology, Tongji Hospital, Frontier Science Center for Stem Cell Research, Shanghai Key Laboratory of Signaling and Disease Research, School of Life Sciences and Technology, Tongji University, Shanghai, China; 2 Department of Pathology, Tongji Hospital, Tongji University School of Medicine, Shanghai, China; 3 Shanghai Pulmonary Hospital, School of Medicine, Tongji University, Shanghai, China; 4 Department of General Surgery, Renji Hospital, School of Medicine, Shanghai Jiao Tong University, Shanghai, China; 5 Division of Life Sciences and Medicine, The First Affiliated Hospital of USTC, University of Science and Technology of China, Hefei, Anhui, China

**Keywords:** malignancy, discoidin domain receptor 1, ubiquitin specific peptidase 7, TP53 mutation, ubiquitination, protein degradation

## Abstract

Receptor tyrosine kinase DDR1 (Discoidin Domain Receptor 1) interacts with the extracellular matrix (ECM) to promote tumor cell proliferation through its intracellular kinase activity, while its extracellular non-enzymatic domain creates a physical barrier for immune evasion. Although DDR1 inhibitors and antibodies have been developed, targeting DDR1 kinase activity alone cannot fully block the biological effects mediated by its scaffold function. Therefore, developing DDR1 degraders presents a potentially more effective therapeutic strategy. Through screening a proprietary small-molecule ubiquitination library, we identified NSC632839, which significantly induces DDR1 protein degradation. Mechanistically, chemical proteomics and genetic studies demonstrated that NSC632839 functions by inhibiting USP7, which interacts with, stabilizes, and deubiquitinates DDR1, preventing its proteasomal degradation. Importantly, we observed that TP53 loss or mutation in tumor cells and clinical samples markedly upregulates DDR1 expression, thereby enhancing its interaction with USP7. Inhibition of USP7 with NSC632839 or other selective inhibitors restores TP53 expression, resulting in a significant reduction in DDR1 levels. In various preclinical models, targeting USP7 with NSC632839 effectively eliminates tumor cells, offering a promising therapeutic strategy to overcome tumor relapse driven by TP53 mutations, both *in vitro* and *in vivo*. This study highlights the potential of DDR1 degradation *via* USP7 inhibition as a novel approach to treat TP53 mutation-enriched tumors.

The discoidin domain receptor 1 (DDR1) belongs to the family of transmembrane receptor tyrosine kinases and is unique for its collagen-binding discoidin domain, distinguishing it from other kinases. Predominantly expressed in epithelial cells, DDR1 plays a key role in matrix remodeling, collagen production and degradation, as well as cellular processes such as proliferation, differentiation, and adhesion (1). It is activated by collagen rather than soluble growth factors (2, 3), triggering its own phosphorylation and activating critical signaling pathways such as MAPK, TGF-β, and Notch (4).

Elevated or mutated DDR1 expression has been observed in various cancer cell lines and primary tumor tissues, including those from the lung (5), pancreas (6), prostate (7), breast (8), brain (9), ovary (10), and liver (11). A recent study revealed that matrix metalloproteinase-cleaved collagen I (cCol I), by binding to DDR1, activates the NF-κB-p62-NRF2 axis, which regulates mitochondrial biogenesis and subsequently promotes the proliferation of pancreatic tumor cells (12). Beyond its intracellular enzymatic role in tumor proliferation, the extracellular non-enzymatic domain of DDR1 has been implicated in promoting immunosuppressive effects within the tumor microenvironment (13). One study demonstrated that DDR1 induces immunosuppression by facilitating the alignment of collagen fibers, which can hinder immune cell infiltration. Additionally, type I collagen (Col I) not only upregulates DDR1 expression but also activates DDR1, promoting the migration and invasion of non-small cell lung cancer (NSCLC) cells. Inhibition of DDR1 in these cells significantly reduces their migratory and invasive potential (5).

In preclinical models, combination therapy using the DDR1 inhibitor dasatinib along with a Notch signaling inhibitor, demcizumab, resulted in enhanced antitumor effects compared to standard chemotherapy. This suggests that targeting DDR1 can disrupt key signaling pathways involved in tumor progression (14). Moreover, recent studies have highlighted that in lung cancer patients who are non-responders to treatment, there is an increased proportion of COL11A1^+^ tumor-associated fibroblasts (CAFs) that interact with tumor cells *via* the DDR1-collagen axis. This interaction creates a physical barrier, limiting T cell infiltration and contributing to immune resistance. These findings suggest that DDR1 may serve as a critical target to overcome immune evasion and improve treatment responses in lung cancer (15). Furthermore, DDR1 overexpression has been observed in a subset of diffuse large B-cell lymphoma (DLBCL), where its expression positively correlates with that of collagen ligands and negatively correlates with the expression of mitotic spindle genes. Together, these findings underscore DDR1's potential as a therapeutic target in a variety of cancers, particularly in those with altered immune responses or collagen-rich tumor microenvironments (16).

TP53 mutations are frequently observed in diffuse large B-cell lymphoma (DLBCL) and refractory/relapsed aggressive B-cell non-Hodgkin lymphoma (r/r B-NHL), where they are strongly associated with poor prognosis, treatment resistance, and relapse (17). In the context of EGFR-mutant non-small cell lung cancer (NSCLC), TP53 loss-of-function mutations have been implicated in early resistance to the EGFR tyrosine kinase inhibitor (TKI) osimertinib (18). Similarly, in B-cell lymphoma, CRISPR/Cas9-based whole-genome screening has identified TP53 deletion as a critical driver of resistance to GSK-591 (19). TP53 mutations undermine apoptosis, rendering tumor cells more resistant to therapies such as chemotherapy and immunotherapy. Notably, DDR1 functions as a direct transcriptional target of TP53 and plays a pivotal role in a positive feedback loop that enhances cell survival in response to DNA damage. Inhibition of DDR1 activity in cells with wild-type TP53 has been shown to promote apoptosis, suggesting that DDR1 inhibition could be a promising therapeutic strategy for tumors with intact TP53. This approach could potentially overcome resistance mechanisms and improve the efficacy of existing therapies in TP53-mutated malignancies (20). Both our study and others have demonstrated that following CAR-T treatment, TP53-mutant lymphoma cells tend to evade immune-mediated cell death, resulting in their survival and contributing to CAR-T relapse (21). These findings collectively highlight DDR1 as a promising therapeutic target in TP53-deficient tumors, offering potential to overcome drug resistance, enhance immune response, and inhibit tumor progression.

Several drugs and antibodies targeting DDR1 are currently in development, with some advancing to clinical trial stages. FDA-approved multi-target small molecule receptor tyrosine kinase inhibitors, such as imatinib, nilotinib, and dasatinib, have demonstrated the ability to inhibit DDR1 kinase activity (22). However, due to the structural homology between ATP-binding pockets of receptor tyrosine kinases, these inhibitors may produce off-target effects. In addition, selective DDR1 inhibitors, including DDR1-IN-1 (23) and 7rh (24), have been developed to specifically target DDR1. Despite their selectivity, these kinase inhibitors primarily target the intracellular enzymatic domain of DDR1, which contributes to tumor proliferation. As a result, these inhibitors are prone to resistance and fail to address the immunosuppressive effects mediated by DDR1’s extracellular non-enzymatic domain, limiting their efficacy in achieving complete tumor regression within the complex tumor microenvironment.

Given the dual roles of DDR1 in tumor progression—where its extracellular non-enzymatic domain exerts immunosuppressive effects, and its intracellular enzymatic domain promotes tumor proliferation—inducing DDR1 degradation may represent a more effective strategy to overcome the limited accessibility of DDR1 inhibitors. This approach could more effectively inhibit tumor growth by targeting both functional domains. The regulation of DDR1 degradation involves a complex interplay of ubiquitin-activating (E1), conjugating (E2), and ligating (E3) enzymes as well as deubiquitylating enzymes (DUBs) (25). Recent research has identified NEDD4L as an E3 ligase responsible for DDR1 degradation, which, in turn, modulates downstream signaling pathways and inhibits the epithelial-mesenchymal transition (EMT) (26). However, the specific DUBs involved in DDR1 regulation remain largely unexplored. To address this gap, we aim to investigate DDR1 degraders and identify DDR1-associated DUBs, offering potential new therapeutic strategies for the treatment of non-small cell lung cancer (NSCLC) and diffuse large B-cell lymphoma (DLBCL).

Here, through screening a small-molecule ubiquitination library, we identified NSC632839, which induces DDR1 degradation by inhibiting USP7. USP7 stabilizes DDR1 by preventing its proteasomal degradation, thereby promoting the proliferation of DDR1-overexpressing NSCLC cells. Additionally, we found that TP53 loss or mutation in DLBCL tumors upregulates DDR1 and enhances its interaction with USP7. Inhibition of USP7 with NSC632839 or other selective inhibitors restores TP53 expression, reduces DDR1 levels, and effectively eliminates tumor cells in patient specimens. Furthermore, targeting USP7 leads to tumor cell elimination in various preclinical models, while also enhancing the cytotoxicity of CAR-T cells against TP53-mutant relapsed/refractory (r/r) DLBCL PDCs. This strategy offers a promising therapeutic approach for TP53 mutation-driven tumors by targeting DDR1 stability *via* USP7 inhibition.

## Results

### Compounds screening identified NSC632839 as a novel DDR1 degrader that effectively inhibits the growth of DDR1-high expressing cells

**Figure 1:**
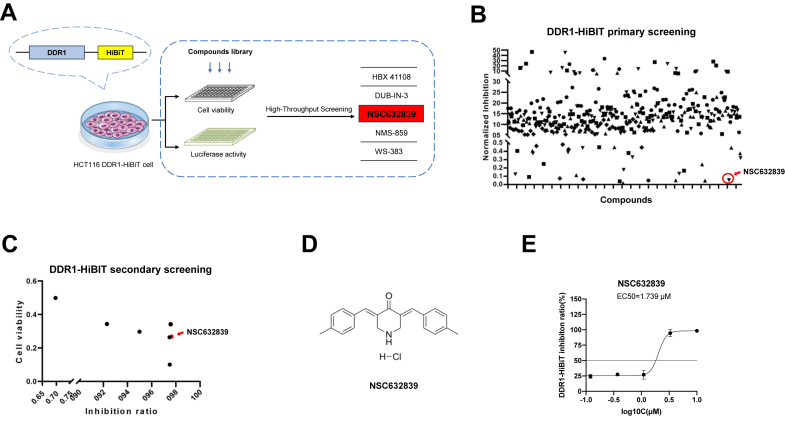
**Compounds screening identified NSC632839 as a novel DDR1 degrader.***A*, the 11-amino acid HiBiT peptide tag was inserted at the C-terminal of the DDR1 protein in HCT116 cells using CRISPR-Cas9 knock-in, enabling real-time tracking and quantification of endogenous DDR1 without compromising its function. Upon cell lysis, the HiBiT-tagged DDR1 protein binds to the complementary LgBiT peptide, forming a luciferase complex that emits fluorescence in the presence of the substrate furimazine. The fluorescence intensity is directly proportional to the amount of HiBiT-tagged DDR1 protein in the cell lysate. Cells were treated with a custom ubiquitination degrader library, and DDR1 degradation was assessed through the HiBiT-luminance assay, allowing visualization of the target effect by Nano-Glo HiBiT Lytic Detection System. *B* and *C*, HCT116 DDR1-HiBiT cells were treated with a library of 368 compounds associated with ubiquitination degradation. The normalized inhibition was visualized by comparing the luciferase signal with the results of the cell viability assay. Through primary and secondary screening, NSC632839 was identified as a top 'hit'. *D*, Chemical structures of NSC632839. *E*, HCT116 DDR1-HiBiT cells were treated with varying concentrations of NSC632839 for 48 h. The inhibition ratio (EC50) was determined by measuring the luciferase intensity in the cells. Mean ± SD. Shown are the representative results of 3 independent experiments.

Given that targeting DDR1 kinase activity alone cannot fully block the biological effects mediated by its scaffold function (12, 13), inducing DDR1 degradation may overcome the limitations of DDR1 inhibitor accessibility, thereby effectively inhibiting the growth of DDR1-high expressing tumor cells. In this study, we first established a DDR1-HiBiT screening cell system using the HCT116 cell line, which highly expresses DDR1. Using CRISPR-Cas9 knock-in technology, we inserted an 11-amino acid HiBiT peptide tag onto the DDR1 protein. This allows for the expression of endogenous DDR1 with a HiBiT tag in the cells. After cell lysis, HiBiT-tagged DDR1 binds to the complementary enzyme LgBiT, generating a luminescent signal upon the addition of furimazine. The intensity of this signal is directly proportional to the amount of HiBiT-tagged DDR1 protein in the cell lysate. We then performed high-throughput screening of a library of 368 compounds associated with ubiquitination degradation using this system. Fluorescence intensity was normalized against cell viability after 24 h of treatment (Fig. 1, *A* and *B*). In the secondary screening, we identified 7 top hits, with NSC632839 exhibiting the strongest DDR1 degradation effect. NSC632839 reduced DDR1-HiBiT protein levels by 97.65% without significantly affecting cell viability (Fig. 1*C*). Treatment with NSC632839 resulted in a dose-dependent degradation of DDR1 protein and selective inhibition of DDR1-high expressing cell growth, with an EC50 value of 1.739 μM, confirming its potency as a DDR1 degrader (Fig. 1, *D* and *E*).

**Figure 2:**
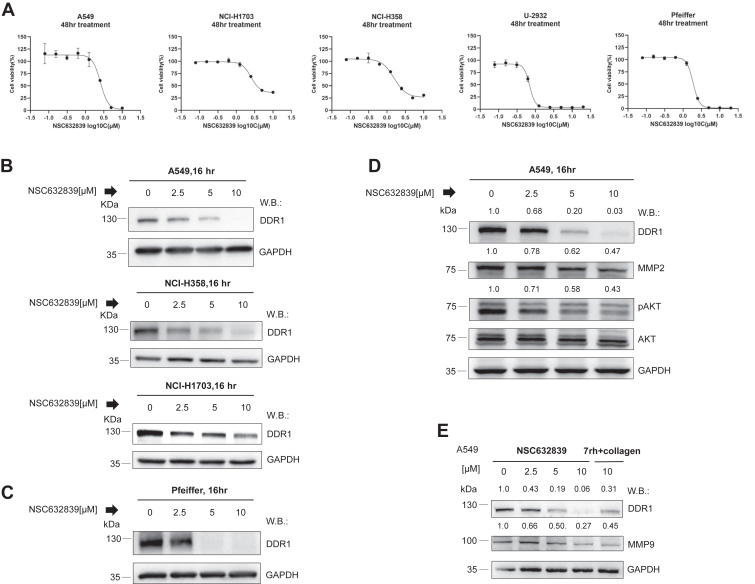
**NSC632839 inhibits the proliferation of malignant tumor cells through degrading DDR1.***A*, the effect of NSC632839 on the proliferation of A549, NCI-H1703, NCI-H358, U-2932, and Pfeiffer cells was assessed using the CellTiter-Glo Luminescent Cell Viability Assay (CTG). Mean ± SD. *B*, impact of NSC632839 on DDR1 expression in A549, NCI-H358, and NCI-H1703 cells. *C*, effect of NSC632839 on DDR1 expression in Pfeiffer cells. *D*, A549 cells were treated with NSC632839 for 16 h at concentrations of 0, 2.5, 5, and 10 μM. DDR1, MMP2, pAKT, AKT, and GAPDH protein levels were assessed by western blotting using the indicated antibodies. *E*, A549 cells were treated with NSC632839 for 16 h at 0, 2.5, 5, and 10 μM, and 7rh at 10 μM. DDR1, MMP9, and GAPDH protein levels were analyzed by western blotting with the indicated antibodies. Shown are the representative results of 3 independent experiments.

We observed that compared to normal tissues, the expression level of DDR1 is significantly upregulated in Lung Adenocarcinoma (LUAD), Lung Squamous Cell Carcinoma (LUSC) and Diffused Large B Cell Lymphoma (DLBCL) (Fig. S1, *A* and *B*). Assessment of Kaplan–Meier curves through immunohistochemistry revealed that high expression of DDR1 is an unfavorable prognostic marker in lung cancer (Fig. S1*C*). We treated the NSCLC cell lines A549, NCI-H1703, and NCI-H358 with NSC632839, and after 16 h, a dose-dependent decrease in DDR1 protein levels and cell viability was observed (Fig. 2, *A* and *B*). Similar results were observed in DLBCL cells U-2932 and Pfeiffer cells (Fig. 2, *A* and *B*). As expected, the degradation of DDR1 in A549 cells correlated with the inhibition of DDR1 downstream signaling pathways, including the phosphorylation of AKT (pAKT) and MMPs (MMP2 and MMP9) (Fig. 2, *D* and *E*). MMPs are key markers for evaluating DDR1’s functional activity (27, 28). Collagen binding activates DDR1’s tyrosine kinase (TYK) activity, triggering downstream signaling (29), which was robustly inhibited by the reported DDR1 inhibitor 7rh (24). Similarly, treatment of A549 cells with 7rh resulted in a comparable reduction in MMP9 signaling, consistent with the effects observed with NSC632839 (Fig. 2*E*). These findings strongly support that NSC632839 inhibits the growth of NSCLC and DLBCL cells by degrading DDR1.

### NSC632839 promotes the ubiquitin-mediated proteasomal degradation of DDR1

**Figure 3:**
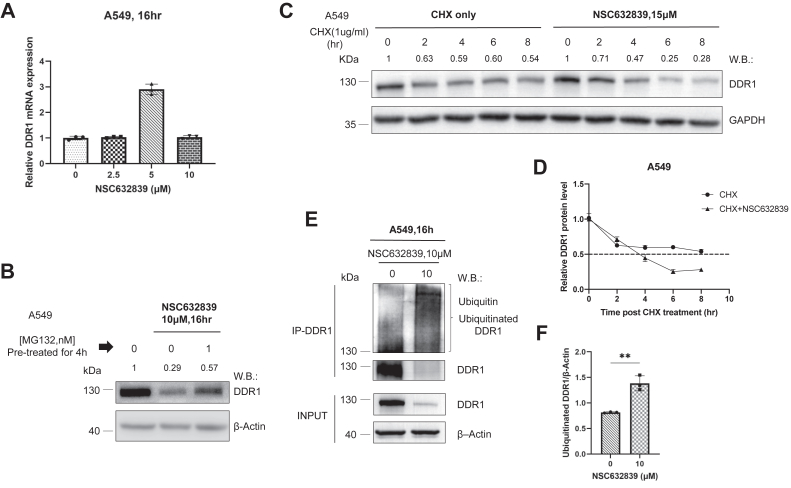
**NSC632839 promotes ubiquitin-mediated proteasomal degradation of DDR1.***A*, A549 cells were treated with NSC632839 for 16 h at concentrations of 0, 2.5, 5, and 10 μM. RNA was isolated, and DDR1 mRNA levels were quantified by qPCR. mRNA expression was normalized to the housekeeping gene GAPDH. Mean ± SD (n = 3). The data represent the results of 3 independent experiments. *B*, A549 cells were pretreated with or without the proteasome inhibitor MG132 (1 nM) for 4 h, followed by 16-h treatment with NSC632839 (10 μM). DDR1 and β-Actin protein levels were detected by immunoblotting. *C* and *D*, A549 cells were treated with cycloheximide (CHX, 1 μg/ml) with or without NSC632839 (15 μM) at the indicated time points. DDR1 and GAPDH protein levels were assessed by immunoblotting. The DDR1 protein half-life was quantified as shown. Mean ± SD. *E* and *F*, immunoprecipitation and Western blotting (I.P./W.B.) were performed to assess the effect of NSC632839 on DDR1 ubiquitination and degradation. The levels of ubiquitinated DDR1 were detected using an anti-ubiquitin antibody, and the data were quantified as shown. Mean ± SD (n = 3); Unpaired *t* test. Data shown are the representative results of 3 independent experiments. ∗*p* < 0.05, ∗∗*p* < 0.01, ∗∗∗*p* < 0.001, ∗∗∗∗*p* < 0.0001.

Building on these results, we sought to investigate whether the observed reduction in DDR1 protein levels was mediated through ubiquitination. To confirm this, we first analyzed the transcriptional levels of DDR1 in A549 cells after NSC632839 treatment. Real-time fluorescent qPCR analysis revealed that the reduction in DDR1 levels occurred solely at the protein level, with no corresponding decrease in transcriptional expression (Fig. 3*A*). Treatment with the proteasome inhibitor MG132 for 4 h, followed by NSC632839 for 16 h, partially rescued DDR1 degradation, suggesting that the compound degrades DDR1 through the ubiquitin-proteasome pathway (Fig. 3*B*). In cycloheximide (CHX) treatments, NSC632839 shortened the half-life of DDR1 protein compared to CHX alone (Fig. 3, *C* and *D*), indicating its effect occurs at the post-translational level. Additionally, immunoprecipitation of DDR1 from A549 cells treated with NSC632839 revealed increased endogenous ubiquitination levels, alongside DDR1 degradation (Fig. 3, *E* and *F*). Collectively, these results confirm that NSC632839 degrades DDR1 through the ubiquitin-mediated proteasomal pathway.

### Mass-spectrum and DUBome identified USP7 as a stabilizing DUB for DDR1

**Figure 4:**
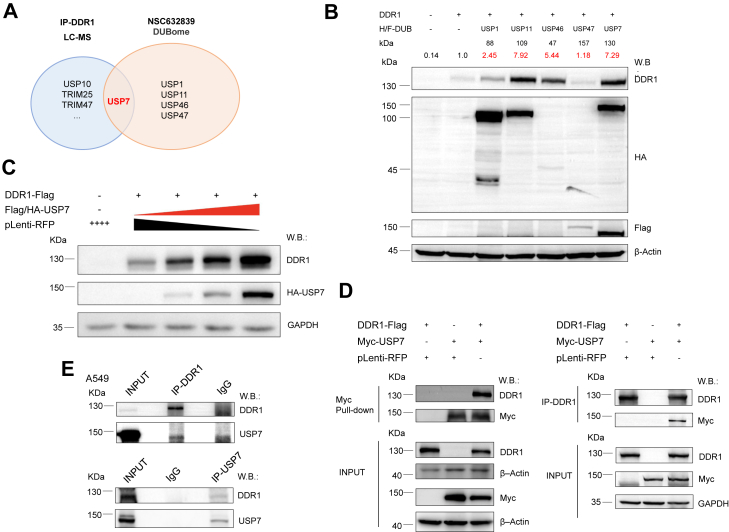
**Mass-spectrum and DUBome identified USP7 as a stabilizing DUB for DDR1.***A*, endogenous DDR1 was immunoprecipitated from A549 cells, followed by mass spectrometry analysis. A DUBome screen of NSC632839 identified potential targets, including USP1, USP7, USP11, USP46, and USP47. USP7 was identified as a common target through the intersection of results from both methods. *B*, co-transfection of potential DUB targets and DDR1 in HEK293 T cells was performed to assess the stabilization effect on DDR1. DUBs were detected using either HA- or FLAG-tag-specific antibodies. *C*, HEK-293T cells were transfected with DDR1-Flag (0.5 μg) and Flag/HA-USP7 (0.5, 1, and 1.5 μg). DDR1 and HA-USP7 expression were detected by immunoblotting using the indicated antibodies. *D*, co-Immunoprecipitation (Co-IP) was performed with Myc IgG beads or DDR1 antibody in HEK-293T cells overexpressing DDR1-Flag and Myc-USP7. DDR1 and Myc-USP7 were detected in both IP and cell lysate (INPUT) by immunoblotting. *E*, reciprocal-co-IP was performed in A549 cells using DDR1 or USP7 antibodies, with IgG as a negative control. DDR1 and USP7 were detected in both IP and cell lysate (INPUT) by immunoblotting. Shown are the representative results of 3 independent experiments.

NSC632839 has been reported as a multitargeted inhibitor, with activity against USP2, USP7, and SENP2 (30). To identify potential DUBs that stabilize DDR1, we performed immunoprecipitation of endogenous DDR1 in A549 cells followed by mass spectrometry analysis, which revealed potential interactions between DDR1 and various ubiquitin-related proteins. On the other hand, we performed an activity-based DUB profiling assay (ABPP) using recombinant DUB proteins and ubiquitin-based activity probes to conduct a DUBome profiling of NSC632839. This analysis identified USP1, USP7, USP11, USP46, and USP47 as potential targets (Fig. S2, *A* and *B*). USP7 was identified as a common target in both the mass spectrometry and the DUBome profiling through the intersection of results from both methods (Fig. 4*A*). Co-transfection experiments in HEK293T cells revealed that USP7 had the strongest stabilizing effect on DDR1 protein compared to other DUBs (Fig. 4*B*, Fig. S2*C*). Additionally, Kaplan-Meier survival analysis across multiple databases showed that high USP7 expression in lymphoma patients is associated with poor prognosis (Fig. S3). This suggests that USP7 may play a critical role in regulating DDR1 expression and could potentially serve as a prognostic biomarker in lymphomas.

We prioritized USP7 as the main candidate and further investigated its interaction with DDR1, along with its stabilizing effect on DDR1 protein levels. Initially, we co-expressed DDR1-Flag and Flag/HA-USP7 plasmids in HEK293 T cells and observed that USP7 dose-dependently stabilized exogenous DDR1 compared to the control group (Fig. 4*C*). To confirm the physical interaction between USP7 and DDR1, we overexpressed DDR1-Flag and Myc-USP7 plasmids in HEK293 T cells. Using magnetic beads conjugated with Myc antibodies or DDR1 antibody-mediated immunoprecipitation (IP) followed by immunoblotting, we detected a robust binding interaction between the 2 proteins (Fig. 4*D*). Additionally, reciprocal-IP assays in A549 cells validated the endogenous interaction between DDR1 and USP7 (Fig. 4*E*). These results collectively support the association between USP7 and DDR1, further corroborating USP7’s role in DDR1 stabilization.

### USP7 deubiquitinates and stabilizes DDR1 to promote tumor cell proliferation

**Figure 5:**
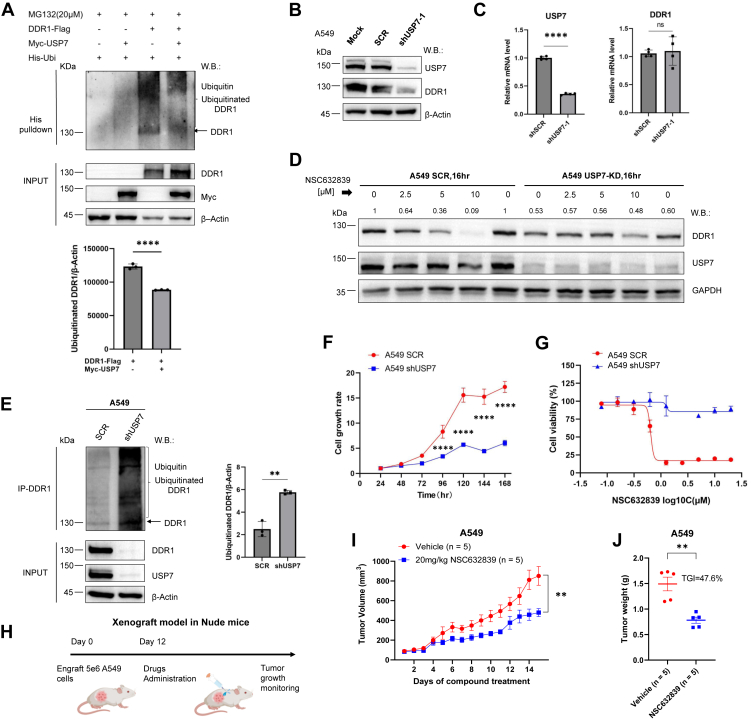
**USP7 deubiquitinates and stabilizes DDR1, thereby promoting tumor cell growth *in vitro* and *in vivo*.***A*, HEK-293T cells were transfected with Flag-tagged DDR1, Myc-tagged USP7, and His-tagged ubiquitin, and treated with MG132 (20 μM) for 4 h prior to collection. Ubiquitinated DDR1 was pulled down using His IgG beads for detection. Ubiquitinated DDR1, USP7, and β-Actin were assessed by western blotting with the indicated antibodies (*upper panel*). The levels of ubiquitinated DDR1 were quantified as shown (*lower panel*). Mean ± SD (n = 3); Unpaired *t* test. *B* and *C*, USP7 was knocked down (KD) in A549 cells using USP7-silencing puromycin-resistant shRNA-1 *via* lentiviral transduction, with a scrambled (SCR) shRNA serving as a non-targeting control. Protein levels of DDR1, USP7, and β-Actin were assessed by western blotting with the indicated antibodies. qPCR was used to measure the mRNA levels of USP7 and DDR1, normalized to GAPDH. Mean ± SD (n = 4); Unpaired *t* test. *D*, A549 SCR and USP7-KD cells were treated with NSC632839 at varying concentrations for 16 h. DDR1, USP7, and GAPDH protein levels were detected by western blotting with the indicated antibodies. *E*, USP7 gene knockdown was performed in A549 cells. DDR1 immunoprecipitation (IP) was carried out, and ubiquitin, DDR1, and β-Actin protein levels were detected in both the IP and cell lysate (INPUT) by western blotting with the indicated antibodies (*left panel*). The levels of ubiquitinated DDR1 were quantified as shown (*right panel*). Mean ± SD (n = 3); Unpaired *t* test. *F*, long-term proliferation effects: Cells were generated as described in (E), and the growth curves of A549-shUSP7 cells were compared to those of SCR control cells using the CTG assay. Mean ± SD; Two-way ANOVA. *G*, short-term cytotoxicity assays: Cells were generated as described in (E), and the immediate effects of NSC632839 on different cell groups were assessed using the CTG assay. *H*, nude mice were implanted subcutaneously with 5e6 A549 cells (n = 5/group), 12 days later, 20 mg/kg (QD) of NSC632839 were given intraperitoneally. *I*, mice were treated with vehicle or NSC632839 (20 mg/kg) and sacrificed on day 15 of treatment. Tumor size measurements of xenograft mice after vehicle and NSC632839 treatment. Mean ± SEM; Two-way ANOVA. *J*, effects of 15 days NSC632839 treatment on growth of xenograft model were determined. Mean ± SEM; Unpaired *t* test. Tumor growth inhibition (TGI) rates were thus determined for both groups using the formula: TGI=((MTVcontrol-MTVtreated)/MTVcontrol) × 100. Shown are the representative results of 3 independent experiments. ∗*p* < 0.05, ∗∗*p* < 0.01, ∗∗∗*p* < 0.001, ∗∗∗∗*p* < 0.0001.

As a deubiquitinating enzyme, USP7 plays a critical role in cleaving and removing ubiquitin chains from substrate proteins (31, 32, 33). To assess its deubiquitinating activity on DDR1, we demonstrated that USP7 effectively removes ubiquitin chains from DDR1, leading to a substantial increase in DDR1 protein levels (Fig. 5*A*). To further investigate the role of USP7 in DDR1 regulation, we performed gene knockdown experiments using shRNA targeting USP7 in A549 cells. Immunoblot analysis revealed a significant reduction in DDR1 levels in USP7 knockdown cells compared to scrambled control cells (Fig. 5*B*). Notably, there was no significant reduction in DDR1 mRNA levels following USP7 knockdown (Fig. 5*C*). To rule out potential off-target effects, we treated SCR and USP7-knockdown cells with NSC632839 for 16 h, results revealed that while depletion of USP7 significantly reduced DDR1 levels, there were no further degradation effects on DDR1 post-NSC632839 treatment (Fig. 5*D*), confirming that NSC632839 targets DDR1 degradation through USP7 inhibition.

To further validate the effect of NSC632839, we performed enzymatic activity assays using purified USP7 protein. The results demonstrated that NSC632839 inhibits USP7 catalytic activity with an IC_50_ of 3.817 μM, which is consistent with the observed DC_50_ for DDR1 degradation in cellular assays (Fig. 2). For comparison, the selective USP7 inhibitor FT671 (34) exhibited an IC_50_ of 286.5 nM under similar experimental conditions (Fig. S4*A*). While these findings highlight the inhibition of USP7 by NSC632839, it is important to note that NSC632839 was primarily employed as a tool compound to probe the USP7-DDR1 regulatory axis rather than a specific USP7 inhibitor. Furthermore, following treatment with FT671, we observed both DDR1 degradation and a reduction in DDR1-positive cell growth, similar to the effects of NSC632839. However, combining FT671 with NSC632839 did not result in additional effects on cell growth, suggesting that NSC632839 induces cell death primarily through USP7 inhibition (Fig. S4, *B–D*).

In line with the increased DDR1 ubiquitination observed upon NSC632839 treatment (Fig. 3, *E* and *F*), USP7 knockdown significantly enhanced the polyubiquitination of DDR1, resulting in pronounced protein degradation (Fig. 5*E*). Furthermore, USP7 knockdown markedly impaired A549 cell proliferation in long-term assays compared to control cells (Fig. 5*F*), consistent with the reduced cell viability observed following NSC632839 treatment (Fig. 2*A*). To assess the immediate cytotoxic effects of NSC632839, we performed a short-term cytotoxicity assay. While USP7 knockdown alone did not significantly affect cell viability in this context, we observed that USP7 knockdown reduced the sensitivity of cells to NSC632839 treatment. This suggests that the cytotoxic effects of NSC632839 are dependent on USP7 inhibition (Fig. 5*G*). To further assess potential off-target effects, we treated both SCR control and USP7-knockdown cells (2 monoclonal lines exhibiting 95% and 85% USP7 knockdown efficiency). We observed that when combined with NSC632839, the 85% knockdown condition showed a clear synergistic effect, while the synergy was less pronounced under 95% knockdown conditions (Fig. S4, *E* and *F*). These results suggest that small residual USP7 activity can influence the efficacy of NSC632839, and that complete USP7 knockdown reduces the synergistic effect. This provides further evidence of NSC632839’s effect and underscores USP7’s critical role in mediating the observed effects.

To further establish a functional link between DDR1 degradation and growth inhibition, we performed DDR1 rescue experiments. Overexpression of DDR1 in the parental cell line, confirmed by Western blot, significantly increased the IC50 of NSC632839, indicating that restored DDR1 expression reversed the compound’s growth-inhibitory effects (Fig. S5, *A* and *B*). Similarly, DDR1 overexpression in USP7 knockdown cells, validated by Western blot, partially rescued the proliferation defect in a 4-day long-term assay (Fig. S5, *C* and *D*). These results support a model in which NSC632839 impairs tumor cell growth primarily through USP7-mediated DDR1 degradation.

We next evaluated the *in vivo* efficacy of NSC632839 against DDR1-positive A549 tumor growth. Female nude mice (5–6 weeks old, n = 5 per group) were subcutaneously implanted with A549 cells and treated with either vehicle or NSC632839 (20 mg/kg, i.p., QD) for the indicated period. Tumor progression was monitored by measuring tumor length and width daily for 15 days post-injection. NSC632839-treated mice developed significantly smaller tumors compared to vehicle controls, with no notable change in body weight (Fig. 5, *H* and *I*, Fig. S6, *A* and *B*). Tumor growth inhibition (TGI) reached 47.6% in the NSC632839 group, indicating a substantial anti-tumor effect (Fig. 5*J*). Additionally, the downstream signaling of DDR1, as evidenced by reduced pAKT levels, was significantly decreased in the NSC632839-treated group (Fig. S6*C*), further highlighting its potential as a therapeutic strategy for DDR1-positive cancers.

Collectively, these findings establish that USP7 deubiquitinates and stabilizes DDR1, and that USP7 inhibition promotes DDR1 ubiquitination and degradation, ultimately suppressing tumor cell proliferation both *in vitro* and *in vivo*.

### TP53 mutation or depletion promotes DDR1 expression and enhances its association with USP7

TP53 mutation is a critical adverse genetic alteration in DLBCL (35), associated with resistance to multiple therapeutic modalities, including conventional immunochemotherapy (36), autologous stem-cell transplantation (37), and CAR-T therapy (21). USP7 plays a complex and dualistic role in regulating TP53 function through intricate mechanisms. Notably, the ablation of USP7 (also known as HAUSP) has been shown to stabilize and activate TP53 (31, 32). Considering the crucial role of USP7 in regulating TP53 and the established finding that DDR1 is a direct transcriptional target of TP53, whose inhibition significantly enhances apoptosis in wild-type TP53 cells in response to genotoxic stress *via* a caspase-dependent pathway (20), the interplay between DDR1 and TP53 in TP53-mutant tumors remains unclear. Furthermore, it has yet to be determined whether targeting USP7 can modulate the regulatory effect of mutant TP53 on DDR1 expression.

**Figure 6:**
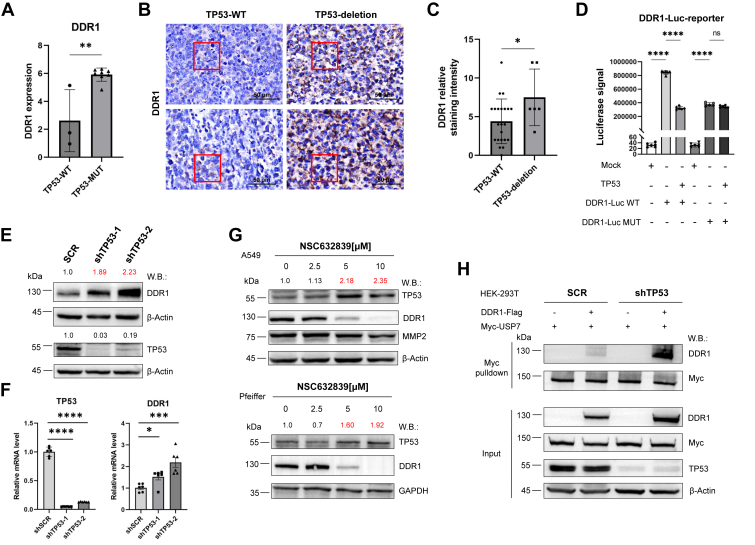
**TP53 mutation or depletion promotes DDR1 expression and its association with USP7.***A*, DDR1 protein levels were quantitatively analyzed in DLBCL cell lines with TP53-WT and TP53-MUT. Mean ± SD; Unpaired *t* test. *B*, tissue microarray (TMA): Tumor tissue samples from 28 DLBCL patients were classified into 2 groups based on *in situ* FISH analysis: TP53-WT (n = 22) and TP53-deletion (n = 6). DDR1 expression was assessed by immunohistochemical staining, with staining intensity (scored from 0 to 3) and positivity rate (scored from 0 to 4) evaluated separately. The composite score, ranging from 0 to 12, was calculated as the product of these 2 scores. Scale bar represents 50 μm. *C*, statistical analysis corresponding to the data shown in panel (B) is presented in this panel. Mean ± SD; Unpaired *t* test. *D*, HEK-293T cells were co-transfected with a DDR1 promoter-driven firefly luciferase reporter containing either wild-type (WT) or mutated (MUT) binding sites, with or without TP53, and incubated for 48 h before harvesting for luciferase activity measurement. Mean ± SD (n = 6); One-way ANOVA. *E* and *F*, TP53 was knocked down in A549 cells using puromycin-resistant shTP53-1 and shTP53-2 lentiviral constructs, with scrambled (SCR) shRNA as a non-targeting control. DDR1, TP53, and β-Actin protein levels were evaluated by western blotting with the indicated antibodies. qPCR was used to measure the mRNA levels of DDR1 and TP53, normalized to GAPDH. Mean ± SD (n = 6); One-way ANOVA. *G*, A549 and Pfeiffer cells were treated with NSC632839 for 16 h at concentrations of 0, 2.5, 5, and 10 μM. DDR1, TP53, MMP2, β-Actin, and GAPDH protein levels were assessed by western blotting using the specified antibodies. *H*, HEK-293T SCR and TP53-KD cells were transfected with Flag-tagged DDR1 and Myc-tagged USP7. Myc pulldown was performed, and DDR1 and Myc protein levels were detected in both immunoprecipitations (IPs) and cell lysates (INPUT) by western blotting using the indicated antibodies. Shown are the representative results of 3 independent experiments. ∗*p* < 0.05, ∗∗*p* < 0.01, ∗∗∗*p* < 0.001, ∗∗∗∗*p* < 0.0001.

Following our prior validation of USP7's role in stabilizing DDR1, we next investigated the relationship between TP53 and DDR1. qPCR analysis of DDR1 protein levels in DLBCL cells revealed significantly higher DDR1 expression in cells harboring TP53 mutations, which are predominantly associated with loss of function (Fig. 6*A*). Tissue microarray (TMA), including immunohistochemistry (IHC) and *in situ* hybridization analysis of patient-derived tissue sections, assessed by staining intensity (0–3 points) and positivity rate (0–4 points), showed that the comprehensive DDR1 scores were significantly elevated in the TP53-deletion group (n = 6) compared to the wild-type TP53 group (n = 22) (Fig. 6, *B* and *C*, Table 1). Notably, we collected peripheral blood samples from 60 patients with DLBCL, including those with either TP53 mutations or wild-type TP53, 7 to 28 days after CAR-T infusion. The samples, which contained both CAR-T cells and a small number of tumor cells, were subjected to transcriptome sequencing. Our analysis revealed a significant increase in DDR1 expression in TP53-mutant patients, particularly those with TP53 deletions. These findings may provide valuable insights into the potential activation of the DDR1 pathway in CAR-T relapse, given DDR1's critical role in immune evasion (Fig. S7*A*). Notably, no significant difference in USP7 expression was observed, suggesting that USP7 may regulate DDR1 and TP53 at the protein level rather than through mRNA expression (Fig. S7*B*, Table 2).

**Table 1:** DLBCL patient characteristics for Fluorescence in Situ Hybridization (FISH) assays and Tissue Microarray (TMA) analysis

Patient	Cytogenetics	Disease type	TP53	DDR1 IRS grade
1	female	DLBCL	negative	8
2	female	DLBCL	negative	3
3	female	DLBCL	negative	6
4	female	DLBCL	negative	6
5	female	DLBCL	negative	2
6	male	DLBCL	negative	6
7	female	DLBCL	negative	2
8	female	DLBCL	negative	1
9	female	DLBCL	negative	2
10	male	DLBCL	negative	0
11	female	DLBCL	negative	4
12	female	DLBCL	negative	2
13	male	DLBCL	negative	6
14	female	DLBCL	negative	8
15	male	DLBCL	negative	2
16	male	DLBCL	negative	6
17	male	DLBCL	negative	6
18	male	DLBCL	negative	1
19	male	DLBCL	negative	6
20	female	DLBCL	negative	12
21	male	DLBCL	negative	4
22	male	DLBCL	negative	4
23	male	DLBCL	positive	6
24	male	DLBCL	positive	12
25	male	DBLCL	positive	3
26	male	DLBCL	positive	6
27	male	DLBCL	positive	6
28	male	DLBCL	positive	12

**Table 2:** DLBCL patient characteristics for transcriptomic analysis

Patient sample	Cytogenetics	Disease type	Mutation type of TP53
A_11_4	Female	DLBCL	MISS
A_7_8	Male	DLBCL	MISS
B_1_1	Female	DLBCL	MISS
B_10_4	Female	DLBCL	MISS
B_11_4	Female	DLBCL	MISS
B_6_1	Male	DLBCL	MISS
B_7_1	Female	DLBCL	MISS
C_4_5	Female	DLBCL	MISS
C_5_5	Male	DLBCL	MISS
C_7_3	Male	DLBCL	MISS
A_1_8	Female	DLBCL	MISS + MUT
A_10_4	Female	DLBCL	MISS + MUT
A_2_8	Male	DLBCL	MISS + MUT
A_4_5	Female	DLBCL	MISS + MUT
B_11_8	Female	DLBCL	MISS + MUT
B_2_1	Male	DLBCL	MISS + MUT
A_12_1	Male	DLBCL	MUT
A_2_4	Male	DLBCL	MUT
A_2_7	Male	DLBCL	MUT
B_1_5	Male	DLBCL	MUT
B_2_8	Female	DLBCL	MUT
B_3_5	Male	DLBCL	MUT
B_5_8	Female	DLBCL	MUT
B_8_5	Male	DLBCL	MUT
C_1_1	Male	DBLCL	MUT
C_2_5	Male	DLBCL	MUT
C_5_4	Male	DLBCL	MUT
C_6_4	Male	DLBCL	MUT
A_10_8	Female	DLBCL	WT
A_11_8	Male	DLBCL	WT
A_12_8	Male	DLBCL	WT
A_3_5	Male	DLBCL	WT
A_6_1	Male	DLBCL	WT
A_6_8	Male	DLBCL	WT
A_7_1	Male	DLBCL	WT
A_7_5	Male	DLBCL	WT
A_8_4	Female	DLBCL	WT
A_9_4	Female	DLBCL	WT
A_9_5	Female	DLBCL	WT
B_10_7	Female	DLBCL	WT
B_12_1	Female	DLBCL	WT
B_12_5	Male	DLBCL	WT
B_3_4	Male	DLBCL	WT
B_4_1	Female	DLBCL	WT
B_4_6	Female	DLBCL	WT
B_5_4	Male	DLBCL	WT
B_6_5	Female	DLBCL	WT
B_7_5	Female	DLBCL	WT
B_8_4	Male	DLBCL	WT
B_9_4	Male	DLBCL	WT
C_1_5	Male	DLBCL	WT
C_4_1	Male	DLBCL	WT
C_6_5	Female	DLBCL	WT
C_6_8	Female	DLBCL	WT
C_7_6	Female	DLBCL	WT
C_8_1	Male	DLBCL	WT
C_8_6	Male	DLBCL	WT
D_5_5	Female	DLBCL	WT
D_6_5	Male	DLBCL	WT
D_6_7	Male	DLBCL	WT

To further explore the impact of TP53 loss on DDR1 expression, we first predicted the TP53 binding sites on the DDR1 promoter using the JASPAR database and constructed a luciferase reporter system driven by the DDR1 promoter in the PGL4 vector. This system included reporters with wild-type (WT) or mutated (MUT) binding sites. HEK-293 T cells were co-transfected with either the WT or MUT DDR1 promoter-driven firefly luciferase reporter, with or without TP53. The results demonstrated that TP53 significantly suppressed transcription of WT DDR1, but this repression was abolished when the binding sites were mutated (Fig. S8*A*, Fig. 6*D*). Subsequent CUT&RUN assays confirmed that TP53 directly binds to the DDR1 promoter, thereby inhibiting its transcription (Fig. S8*B*). Furthermore, NSC632839 treatment significantly enhanced TP53-mediated transcriptional repression of DDR1. This indicates that, in addition to promoting DDR1 degradation through USP7 inhibition, NSC632839 upregulates TP53, which synergistically suppresses DDR1 transcription and further reduces DDR1 levels (Fig. S8, *C* and *D*). Next, we performed TP53 knockdown in HEK293 T cells as well as wild-type TP53 A549 cells, observing a notable increase in DDR1 protein levels (Fig. S8*E*, Fig. 6*E*). Additionally, qPCR analysis revealed that TP53 knockdown significantly elevated DDR1 transcription, suggesting that the loss of TP53 drives DDR1 overexpression (Fig. 6*F*). Treatment of A549 and Pfeiffer cells with NSC632839 inhibited USP7, leading to increased TP53 levels and decreased DDR1 protein levels (Fig. 6*G*). Additionally, co-expression of DDR1-Flag and Myc-USP7 in control and TP53-knockdown cell lines revealed that TP53 deletion not only elevated DDR1 protein levels but also significantly enhanced the interaction between DDR1 and USP7 (Fig. 6*H*). Specifically, we acknowledge that the observed enhanced interaction between DDR1 and USP7 may primarily be a consequence of increased DDR1 expression rather than an intrinsic strengthening of their binding. Thus, our model suggests that TP53 loss leads to DDR1 upregulation, providing more DDR1 protein available for interaction with USP7, rather than implying an inherent increase in the strength of their interaction. These findings propose that TP53 loss promotes DDR1 expression and enhances its interaction with USP7, which could play a role in tumor survival and contribute to resistance mechanisms in TP53-mutant tumors.

### NSC632839 leads to killing of TP53 mutant-DDR1-positive PDCs both *ex vivo* and *in vivo*

**Figure 7:**
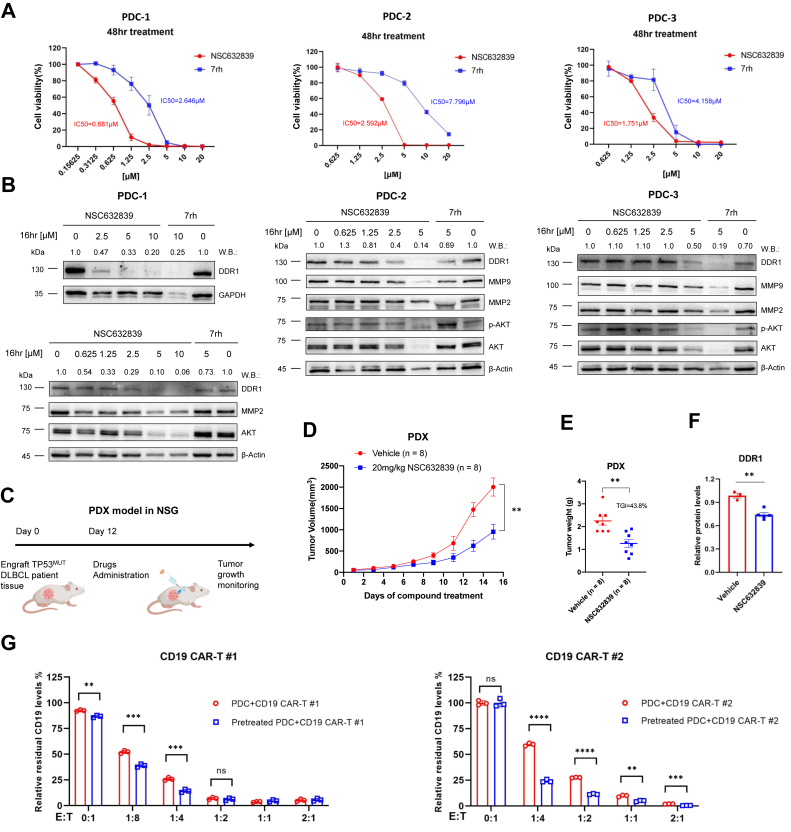
**NSC632839 leads to the killing of TP53-mutant DDR1-positive PDCs both *ex vivo* and *in vivo*.***A*, patient-derived cells (PDCs) from DLBCL patients (n = 3) were treated with NSC632839 or 7rh at varying concentrations for 48 h. Cell proliferation was assessed using the CellTiter-Glo (CTG) assay. Mean ± SD. *B*, PDCs were treated with NSC632839 or 7rh for 16 h, followed by cell lysis to detect protein levels of DDR1, MMP9, MMP2, p-AKT, AKT, β-Actin, or GAPDH. The data shown are representative of 3 independent experiments. *C*, NSG mice were implanted subcutaneously with TP53^MUT^ DLBCL patient tissue (n = 8/group), 12 days later, 20 mg/kg (QD) of NSC632839 was given intraperitoneally. *D*, mice were treated with vehicle or NSC632839 (20 mg/kg) and sacrificed on day 16 of treatment. Tumor size measurements of PDX mice after vehicle and NSC632839 treatment. Mean ± SEM; Two-way ANOVA. *E*, effects of 16 days of NSC632839 treatment on the growth of the PDX model were determined. Mean ± SEM; Unpaired *t* test. Tumor growth inhibition (TGI) rates were thus determined for both groups using the formula: TGI = ((MTVcontrol-MTVtreated)/MTVcontrol) × 100. *F*, DDR1 protein levels in tumor tissue were determined by western blotting, followed by quantification and representation in a bar graph. Mean ± SEM; Unpaired *t* test. *G*, CD19 CAR-T cells were generated from human pan-T cells isolated from PBMCs using a human pan-T isolation kit. The T cells were activated with CD3/CD28 cross-linking antibody beads and transduced with CAR lentiviral constructs at an MOI of 5 to 10 on day 3. Following transduction, the cells were expanded *in vitro* and co-cultured with primary TP53-mutant DLBCL PDCs (with or without NSC632839 pretreatment for 16 h at concentration of 2.5 μM) at E:T ratios ranging from 1:8 to 2:1. The co-culture was maintained for 4 h. The remaining CD19 levels were analyzed by flow cytometry using the indicated antibodies. Mean ± SD (n = 3); Two-way ANOVA. Shown are the representative results of 3 independent experiments. ∗*p* < 0.05, ∗∗*p* < 0.01, ∗∗∗*p* < 0.001, ∗∗∗∗*p* < 0.0001.

Given that TP53 mutation enhances DDR1 expression and its interaction with USP7, we next evaluated the cytotoxic effects of NSC632839 on TP53-mutant DDR1-positive cells. Patient-Derived Cells (PDCs) from TP53-mutant DLBCL patients (n = 3) were treated with NSC632839 or 7rh (positive control), followed by an assessment of cell proliferation. Detailed patient information is provided in Table 3. After 48 h of treatment, NSC632839 exhibited superior inhibition of cell proliferation compared to 7rh, with a significantly lower half-maximal inhibitory concentration (IC50) (Fig. 7*A*). Additionally, the selective USP7 inhibitor FT671 induced significant cytotoxicity in PDCs (Fig. S9), highlighting USP7 targeting as a promising therapeutic strategy for TP53-mutant, r/r DLBCL. Further analysis of DDR1 protein levels and associated downstream signaling pathways revealed that NSC632839 reduced DDR1 expression and its downstream signaling in a dose-dependent manner, indicating that the compound suppresses PDC cell proliferation through DDR1 degradation (Fig. 7*B*).

**Table 3:** TP53 mutant-DLBCL primary patient PDC sample characteristics

Primary patient sample	Age	Disease type	Cytogenetics	Mutations	Abundance	Mutation types
Patient 1	67	DLBCL	46,XY	TP53 NM_000546 exon 7 c.749 C>, T p.P2501	100%	Missense mutation
Patient 2	28	DLBCL	46,XX	TP53 NM_000546 exon 8, p.R273 C, c.817 C > T, p.Arg273Cys; TP53 NM_000546 exon 7, p.Y236 N, с.706 T > A, p.Tyr236Asn; TP53 NM_000546 exon 9, c.993+2T > A	45.63%; 42.19%; 3.05%	Missense mutation; Missense mutation; Alternative splicing mutation
Patient 3	42	DLBCL	46,XY	TP53 NM_000546 exon	100%	Missense mutation

Given that NSC632839 specifically destabilizes DDR1 by targeting USP7 *in vitro* and *ex vivo*, and demonstrates significant *in vivo* efficacy in the A549-CDX model, we further evaluated its impact on the growth of TP53-mutant, DDR1-positive DLBCL *in vivo*. Subcutaneous (s.c.) implantation of DLBCL patient tissue (Patient 1, n = 8 mice/group) was performed in female NCG mice (6–8 weeks old), followed by treatment with either vehicle or NSC632839 (20 mg/kg, i.p., QD) for the indicated durations. Detailed patient information is provided in Table 3. Tumor development and progression were monitored by measuring tumor length and width every 2 days for 16 days post-injection. NSC632839-treated mice exhibited smaller tumors than vehicle controls, with no evident signs of toxicity (Fig. 7, *C* and *D*, Fig. S10). Tumor growth inhibition (TGI) was significantly greater in NSC632839-treated mice (43.8%) compared to controls (Fig. 7*E*). Analysis of DDR1 protein levels in excised tumors is shown in Figure 7*F*. As both our study and others show, TP53-mutant lymphoma cells evade immune-mediated cell death, contributing to CAR-T relapse (21). Given that these TP53-mutant PDC samples are CD19^+^, we conducted CAR-T co-culture experiments with PDCs pretreated with NSC632839 for 16 h to better highlight the clinical relevance of our findings. These experiments demonstrated enhanced cytotoxicity across E:T ratios of 1:8 to 2:1, further emphasizing the role of USP7 inhibition in TP53-mutant r/r cancers (Fig. 7*G*). These results are consistent with the effects observed in mutant DLBCL cells in liquid proliferation assays (Figs. 2, 7, *A* and *B*, Fig. S9).

Taken together, the potent cytotoxicity observed in various TP53-mutant DLBCL models underscores the therapeutic potential of USP7 inhibition in targeting DDR1, offering a promising approach for the treatment of TP53-mutant r/r DLBCL.

## Discussion

DDR1 is a critical driver of tumorigenesis, contributing through both its intracellular kinase activity and extracellular non-enzymatic functions, positioning it as an important target for cancer therapy. Although compounds like imatinib (38), nilotinib (39), and dasatinib (39) have been identified as DDR1 inhibitors, their lack of specificity—stemming from their original design to target ABL kinases—limits their effectiveness, particularly within the complex tumor microenvironment. Furthermore, resistance to DDR1 inhibitors can develop as tumor cells reactivate DDR1-related signaling pathways (40). Efforts are underway to develop more selective DDR1 inhibitors and monoclonal antibodies, such as DDR1-IN-1 (23), 7rh (24) and 48B3 (41), which have demonstrated promise in preclinical studies by inhibiting tumor growth. However, current small-molecule inhibitors and antibodies primarily target DDR1's kinase activity or extracellular domain, failing to fully disrupt its involvement in tumor progression. In contrast, DDR1 degraders, by promoting the proteasomal degradation of DDR1, offer a more comprehensive therapeutic strategy, effectively eliminating both its enzymatic and scaffold functions and providing a more potent approach for cancer treatment.

In this study, we identified NSC632839 as a novel DDR1 degrader through chemical genetics screening. This compound effectively targets both the intracellular enzymatic and extracellular non-enzymatic functions of DDR1, potentially addressing key challenges such as poor inhibitor specificity, drug resistance, and limited accessibility. While our study primarily focuses on DDR1-high expressing NSCLC and TP53 mutant DLBCL, DDR1 expression is significantly elevated in 17 other cancer types, suggesting that NSC632839 holds potential as a therapeutic strategy for a broader range of DDR1-associated cancers. However, further investigation is required to evaluate its *in vivo* antitumor efficacy, pharmacokinetic profile, and potential for synergistic effects when combined with other treatment modalities, such as platinum-based chemotherapeutic agents.

While NSC632839 is a known PAINS compound with potential off-target reactivity (42), our ABPP-based DUBome analysis identified USP7, along with USP1, USP11, USP46, and USP47, as the primary targets. However, through a combination of overexpression experiments and mass spectrometry, we pinpointed USP7 as the key mediator of DDR1 degradation. Further validation using USP7 KD or the USP7-specific inhibitor FT671 confirmed that neither approach, when combined with NSC632839, resulted in additional effects on DDR1 degradation or cell growth inhibition, demonstrating that NSC632839 exerts its effects specifically through USP7. It is important to note that in this study, NSC632839 was used as a tool compound to probe the role of USP7 in DDR1 stability rather than as a therapeutic candidate. We acknowledge that while NSC632839 was effective in our study, its specificity remains a limitation, and future studies will focus on developing more selective inhibitors of USP7. Nevertheless, our work highlights the potential of targeting USP7 for therapeutic intervention, particularly in TP53 mutation-driven cancers, where it may offer a promising strategy to overcome resistance and enhance treatment efficacy.

Additionally, while NEDD4L has been identified as an E3 ligase responsible for DDR1 ubiquitination, the role of DUBs in DDR1 regulation remains poorly understood. Through a combination of DUBome analysis and proteomics, we discovered USP7 as a novel DUB that stabilizes DDR1 by mediating its ubiquitin-dependent stabilization. To our knowledge, USP7 is the first DUB identified in the regulation of DDR1, highlighting its critical role in maintaining DDR1 stability. Elevated USP7 expression in tumors is frequently associated with metastasis, drug resistance, and poor prognosis (43, 44). The identification of USP7 as a key regulator of DDR1 and the potential development of USP7 inhibitors open new therapeutic avenues for targeting the USP7-DDR1 axis in cancer treatment. While our current data strongly suggest that USP7 modulates DDR1 ubiquitination and stability, the direct enzymatic relationship between USP7 and DDR1 remains to be conclusively established. Future studies utilizing purified components in *in vitro* deubiquitination assays will be required to validate DDR1 as a *bona fide* direct substrate of USP7. Furthermore, USP7’s prognostic significance likely arises from its regulation of multiple substrates, including DDR1, underscoring its complex role and potential as a prognostic marker in various malignancies.

TP53 mutations are widely recognized as pivotal genetic alterations that drive tumorigenesis, particularly in relapsed and drug-resistant lymphomas. Studies have also identified TP53 mutations as key contributors to relapse in diffuse large B-cell lymphoma (DLBCL) (17, 18, 19, 20, 21). However, the precise mechanisms underlying this process and the development of effective therapeutic strategies remain poorly understood. In this study, we demonstrate that TP53 mutations and deletions lead to significantly elevated DDR1 levels in clinical DLBCL samples, enhancing its interaction with USP7. Specifically, luciferase reporter assays and CUT&RUN experiments provide evidence that TP53 binds to the DDR1 promoter and suppresses its transcription. This suggests a distinct regulatory mechanism in hematologic malignancies, with p53 regulation of DDR1 differing from its role in solid tumors. We further show that inhibiting USP7 activity with small molecules restores TP53 expression and reduces DDR1 levels, offering a dual therapeutic approach to target TP53-driven relapsed tumors. Moreover, in TP53-mutant r/r DLBCL PDCs, pre-treatment with the USP7 inhibitor significantly boosted the subsequent cytotoxicity of CD19 CAR-T cells. This strategy holds promise for improving treatment outcomes in TP53-mutant lymphomas.

The observed increase in DDR1 expression following TP53 mutation or loss in DLBCL contrasts with findings in solid tumors, where DDR1 is typically upregulated by wild-type TP53(20). This discrepancy may reflect differences in cellular context, chromatin accessibility, or co-factor availability, and suggests that DDR1 may act as an oncogenic driver downstream of TP53 loss in certain cancers. These findings highlight the complexity of TP53's role in regulating DDR1 and propose a novel mechanism by which TP53 mutations promote tumor progression in DLBCL.

The combination of DDR1 degraders and inhibitors presents a promising therapeutic strategy by simultaneously targeting both the enzymatic and scaffold functions of DDR1. While DDR1 inhibitors primarily block its kinase activity, DDR1 degraders reduce the total protein levels of DDR1, thereby addressing its roles in both signaling and structural processes that contribute to tumor progression and immune evasion. This dual approach offers significant potential in overcoming resistance mechanisms and enhancing tumor suppression, particularly in TP53-mutant cancers. Furthermore, the synergistic use of DDR1 degraders with chemotherapy, other targeted therapies, or immunotherapy (including cell-based therapies) could improve treatment efficacy by sensitizing tumors to therapeutic agents, disrupting the tumor microenvironment, and reinstating immune responses. This integrated approach provides a comprehensive strategy for targeting DDR1-driven malignancies and holds substantial promise for advancing clinical applications.

Taken together, our findings identify USP7 as a novel stabilizer of DDR1 through chemical genetics screening and establish a critical link between TP53 mutation-driven tumors and DDR1. These insights shed light on the mechanisms underlying relapse in TP53-mutant cancers. By targeting DDR1 stability *via* USP7 inhibition, we propose a new therapeutic strategy with significant clinical implications for TP53-mutated malignancies. Moreover, our results highlight the potential of targeting both the enzymatic and scaffold functions of DDR1 to enhance therapeutic efficacy, particularly in TP53-mutant tumors. This integrated approach offers a promising strategy to overcome resistance mechanisms and improve treatment outcomes in cancers characterized by TP53 mutations and DDR1 dysregulation.

## Experimental procedures

### Cell lines and cell culture

All cell lines utilized in this study were cultivated in a 5% CO2 atmosphere at 37 °C. The human non-small cell lung cancer cell line A549 was propagated in F-12K medium (Pricella Biotechnology), which was enriched with 10% fetal bovine serum (FBS, VivaCell C04001) and 1% penicillin/streptomycin. 293T cells, human non-small cell lung cancer cell line H358, and human lung squamous carcinoma cell line H1703 were cultured in DMEM high glucose medium (Gibco), also containing 10% fetal bovine serum (FBS, VivaCell C04001) with 1% penicillin/streptomycin. Human diffuse large B-cell lymphoma cells, Pfeiffer, Karpas422, U-2932, and human B-lymphoma cell line Farage were cultivated in RPMI1640 medium (Gibco, VivaCell C04002), with the remaining conditions being identical to those described above. All of the human cell lines listed above were >80% matches to the cell lines listed in the STR of the ATCC Cell Line Repository.

### Antibodies and reagents

All antibodies were used at 1:1000 for immunoblotting, with the exception of anti-GAPDH and anti-β-Actin, which were used at 1:5000. The following antibodies were purchased from Proteintech: MMP2 (10373-2-AP), MMP9 (10375-2-AP), AKT (60203-2-Ig), FLAG-tag (66008-4-Ig), His-tag (66005-1-Ig), TP53 (10442-1-AP), GAPDH (60004-1-Ig), β-Actin (66009-1-Ig). DDR1 (5583T, 5583S) and pAKT (4060S) antibodies were purchased from Cell Signaling Technology (CST). Myc-tag (M20002) antibody was purchased from Abmart. All of the primary antibodies above are described on the manufacturer's website and their application validation.

Protein A/G and Myc magnetic beads were purchased from MedChemExpress (MCE). The CellCounting-Lite 2.0 Luminescent Cell Viability Assay kit was obtained from Vazyme (DD1101–02). The Nano-Glo HiBiT Lytic Detection System (N3040) was sourced from Promega. Immobilon Western HRP substrate was purchased from Millipore (WBKLS0500). Buffer solutions were prepared using the pH adjustment calculator from Mettler Toledo (FE28-Standard).

### Chemical compounds

The ubiquitination degrader library, comprising 368 compounds, was partially sourced from MedChemExpress and Targetmol, with the remaining compounds designed and synthesized in-house to form a custom mixed collection. This library includes both literature-reported DUB/E3 ligands and novel compounds designed and optimized in our lab. NSC632839 and 7rh were purchased from Targetmol. Inhibitors were dissolved in DMSO to obtain 10 mM stock solutions. Serial dilutions were then made to obtain final dilutions for cellular assays with a final concentration of DMSO between 0.2 and 0.5%. Cycloheximide (CHX) and MG132 were purchased from Sigma-Aldrich.

### Immunoblotting and immunoprecipitation

RIPA buffer from NCM (main components: 50 mM Tris (pH 7.6), 150 mM NaCl, 1% NP-40, 0.5% sodium deoxycholate, 0.1% SDS) with an additional 1% protease inhibitor was used as the lysate. Cells were treated with 0, 2.5, 5, and 10 μM NSC632893 for 16 h. Cells were collected and treated with the lysate, and levels of DDR1 and downstream signaling proteins were detected by immunoblotting with the indicated antibodies.

For immunoprecipitation, at least 1 mg of protein per sample was prepared, and at least 1 μg of antibody was used. IgG was used as a non-targeting control.

### Ubiquitin pull-down assay

HEK 293T cells transfected with target proteins, DUB and His-tagged ubiquitin were harvested by treatment with 20 μm MG132 for 4 h. Cells were rinsed with ice-cold 1 × PBS and lysed in pH 8.0 urea buffer (10 mM Tris at pH 8.0, 8 M urea, 100 mM Na_2_HPO_4_, 0.2% Triton-100, 1 mM N-ethylmaleimide and 10 mM imidazole) for 30 min. The lysate was incubated with 10 μL Anti-His beads (HY-K0209, MCE) for 3 h at room temperature. Before incubation, the beads were washed 3 times with pH 8.0 urea buffer. After 3 h incubation, the beads were washed 3 times with pH 8.0 urea buffer, pH 6.3 urea buffer (10 mM Tris, 8 M urea, 100 mM Na2HPO4, 0.2% Triton-100 and 10 mM imidazole, pH 6.3) and wash buffer (20 mM Tris, 100 mM NaCl, 20% glycerol, 1 mM dithiothreitol and 10 mM imidazole, pH 8.0) were washed twice each. Beads were boiled for 5 to 10 min in 30 μl 2 × sample buffer, separated on SDS-PAGE gels, and the ubiquitination levels were detected by Western blotting using the indicated specific antibodies. Specifically, the levels of ubiquitinated DDR1 were detected using an anti-ubiquitin antibody.

### Knockdown (KD) assay

The puro-resistant lentiviral shRNA vector particles against USP7 and TP53 were designed and prepared in-house. Cells were incubated with the viral particles in the presence of 5 μg/ml Polybrene for 24 h and then replaced with fresh medium. After 72 h, the cells were screened with 0.5 to 1 μg/ml puromycin for 72 h.

USP7 KD studies in A549 cells: Vectors containing scramble (SCR) shRNA as a control or USP7 shRNA were co-transfected with psPAX2 and pMD2.G. The resulting viral particles were concentrated by incubation in 5 × PEG. A549 cells were then infected in the presence of 5 μg/ml Polybrene, and selection was initiated with 0.5 to 1 μg/ml puromycin 48 h post-infection. Monoclonal cell selection: Cells were diluted to a concentration of 50 cells per 10 ml of complete medium, mixed thoroughly, transferred to 96-well plates (flat) and incubated in a 5% CO2 incubator at 37 °C for 1 to 2 weeks.

TP53 KD studies in HEK-293T, A549, and Pfeiffer cells were similar to those described above. The shTP53-1, shTP53-2 sequences were purchased by Human Fenghui Biotechnology Co., Ltd. The sequences of the shRNA are as follows.

**Table:** 

Name	Target sequences
shUSP7-1	TTGTGGTTACGTTATCAAATA
shTP53–1	GAGGGATGTTTGGGAGATGTA
shTP53–2	CACCATCCACTACAACTACAT

### Cell transfection

The HEK293 T cells were cultured in DMEM containing 10% FBS, at 37 °C with 5% CO_2_. Cells were seeded at a density of 20,000 cells/well in a 24-well plate 1 day prior to transfection and were transfected with Polyethylenimine (PEI) (40816ES02, Yeasen) according to the manufacturer's instructions. After 6 h, the transfection medium was replaced with fresh DMEM. The plasmid used were DDR1-Flag, HA/Flag-USP1, HA/Flag -USP2, HA/Flag -UCHL5, HA/Flag -USP7, HA/Flag -USP11, pLenti-RFP, Myc-USP7, and His-ubiquitin.

### Quantitative real-time polymerase chain reaction (qPCR)

mRNA was extracted using the HiPure Total RNA Mini Kit (R4111, Magen), followed by reverse transcription using the Strand cDNA Synthesis SuperMix (11123ES10, Yeasen). Quantitative real-time PCR (qRT-PCR) was performed in a 96-well plate with SYBR Green Master Mix (11201ES03, Yeasen). GAPDH served as the internal control for normalizing gene expression, and relative expression levels were calculated using the 2-ΔΔCt method.

The sequences of primers are as follows:

DDR1-Foward: AAGGGACATTTTGATCCTGCC.

DDR1-Reverse: CCTTGGGAAACACCGACCC.

USP7-Forward: GGAAGCGGGAGATACAGATGA.

USP7-Reverse: AAGGACCGACTCACTCAGTCT.

GAPDH-Forward: GGAGCGAGATCCCTCCAAAAT.

GAPDH-Reverse: GGCTGTTGTCATACTTCTCATGG.

### PDC liquid culture and proliferation assays

Patient-derived tumor tissues were obtained from Tongji Hospital. The tissues were immersed in a digestion solution at a ratio of 5 to 10 times the tissue volume. The digestion solution was prepared using Hank's Balanced Salt Solution (HBSS) as a solvent and contained 0.05 wt% collagenase type IV, 0.001 wt% DNase I, and 0.111 g of CaCl_2_ per 200 ml of HBSS. The tissues were incubated in this solution for 3 h. After incubation, the tumor tissue was trimmed into small fragments (∼0.5 mm^3^) and transferred to culture dishes. The fragments were then digested in a trypsin-containing solution in a 37 °C water bath with shaking for 1 to 2 h to achieve complete dissociation of the tumor tissue.

The resulting cell suspension was filtered through a 200-mesh metal sieve and cultured in RPMI 1640 medium (Gibco) supplemented with 10% fetal bovine serum (FBS, VivaCell C04001) and 1% penicillin/streptomycin. The tissue cryopreservation solution (PRS-OCR) was purchased from Hefei PreceDo Medical Laboratory Co., Ltd. For viability assays, 10,000 cells per well were seeded into 96-well plates and incubated with a gradient of NSC632839 or 7rh concentrations for 48 h. Cell viability was assessed using the CellTiter-Glo Luminescent Cell Viability Assay.

### Mass spectrometry-based proteomics

DDR1 proteins are extracted from A549 cells and followed by enzymatic digestion, which cleaves proteins into smaller peptides. DDR1 peptides are separated using liquid chromatography, which helps to reduce sample complexity by separating peptides based on physicochemical properties. Then the peptides are introduced into the mass spectrometer. The ionization process generates charged peptides. The ionized peptides are analyzed in the mass spectrometer, and then the data collected provides information about the peptide’s molecular weight and sequence. The quantitative mass spectrometry was performed and analyzed by Omics Space. The raw mass spectrometry data generated have been deposited in the PRIDE public repository under accession code: PXD065332. Token: Wp9QjscIIVgg.

### ABPP-DUBome

Comprehensive selectivity profiling of NSC632839 was conducted using an activity-based DUB profiling assay (ABPP), incorporating both purified enzyme biochemical assays and chemical proteomics, as previously described (45, 46). Briefly, we analyzed the compound’s selectivity against a panel of 41 purified DUB enzymes, using ubiquitin-AMC as the substrate. This screening identified USP7, USP11, USP2, and USP1 as the key targets. The raw data generated have been deposited in the MassIVE public repository under accession code: MSV000088637 [10.25345/C51K2F].

### Ubiquitin AMC assay

Fluorescence intensity measurements were used to monitor the cleavage of a Ubiquitin-AMC substrate. The activity assays were performed in black 384-well plates (Greiner Bio-One). The recombinant human-derived USP7 protein (purchased from MedChemExpress) was tested for activity in the Ubiquitin-AMC assay in the presence or absence of inhibitors. For this assay, 2 nM USP7 was pre-incubated with different concentrations of inhibitors or DMSO as a control in 50 mM Tris pH 8.0, 100 mM NaCl, 1 mM DTT. The reaction was incubated for 0.5 h at room temperature prior to the addition of 1 μM Ubiquitin-AMC substrate. The reaction was incubated for 1 h before measuring fluorescence intensity on a Multimode Microplate Reader (Tecan Spark) with a 345 nm excitation/445 nm emission. Fluorescence values were normalized to DMSO controls. Dose-response curves were generated using GraphPad Prism.

### DLBCL patient cells

PDC-1, PDC-2, and PDC-3 cells were isolated from DLBCL patient samples and cultured in liquid culture (RPMI 1640 supplemented with 10% FBS) in the presence of the drug. All patient-derived cells (PDC) were obtained under the approval of the Tongji Hospital Review Board. The patient’s information is provided in Table 3.

### Fluorescence in situ hybridization (FISH) and tissue microarray (TMA) analysis

Tumor tissue samples from 28 DLBCL patients were classified into 2 groups based on *in situ* FISH analysis: TP53-WT and TP53-deletion. Briefly, FISH assays were performed on the tissue sections according to operating instructions (Guangzhou LBP Medicine Science & Technology Co., Ltd). Assays were completed using the probe to detect TP53 gene deletion. The FISH results were analyzed by fluorescence microscopy (BX51, Olympus, Tokyo, Japan). Concurrent controls were run for each of the probes tested. Among 100 analyzed nuclei, more than 20% of tumor cells showed a positive co-localization pattern of one red fluorescent signal (indicating TP53 deletion) and 2 green fluorescent signals (indicating CSP17 expression), consistent with the expected pattern for TP53 deletion.

Small cylindrical tissue cores (1.5 mm in diameter) are extracted from formalin-fixed, paraffin-embedded (FFPE) tumor tissues and arranged in a grid pattern on the paraffin block. These tissue cores are precisely positioned using a specialized automated tissue arrayer. The 3 μM thick slides sectioned from the TMA paraffin block were then subjected to various analyses, including immunohistochemistry (IHC) and *in situ* hybridization, to study DDR1 and TP53 protein expression, gene alterations and histopathological features across different tumor samples. The clinical characteristics of these DLBCL patients can be found in Table 1.

### DLBCL blood samples for transcriptomic analysis

Peripheral blood samples were collected from 60 DLBCL patients post-CAR-T infusion with approval from the Tongji Hospital Review Board. The clinical characteristics of these patients are provided in Table 2. The bulk-seq was carried out by Novogene Co., Ltd following the manufacturer’s instructions. The raw data generated in this study have been deposited in the GEO database under accession number GSE301195. Please use the token ohkpugicvjktpyt to access the data.

### Animal study

#### A549-cell line-derived xenograft model

Female SCID nude mice (5–6 weeks old) were obtained from Beijing Vital River Laboratory Animal Technology Co., Ltd. Prior to implantation, exponentially growing A549 cells were harvested and resuspended in DMEM medium. A suspension containing 5 × 10^6^ cells was mixed 1:1 with Matrigel (BD Biosciences) and inoculated subcutaneously into the right flank of each mouse. Treatment commenced when tumor volumes reached 100 to 150 mm^3^, at which point the mice were randomized into experimental cohorts (n = 5 mice/group). For therapeutic evaluation, NSC632839 was administered daily *via* intraperitoneal injection at a dose of 20 mg/kg, formulated in a vehicle consisting of 5% DMSO and 95% HKI solution (0.5% Methocel/0.4% Tween 80 in ddH_2_O). Control groups received vehicle alone. Tumor progression and body weight were monitored daily post-treatment. Tumor volume (mm^3^) was determined using the formula: V = (W^2^ × L)/2, where W (width) and L (length) represent the shortest and longest diameters, respectively.

#### TP53^MUT^-patient-derived xenograft model

Murine tumor xenograft models were performed as previously described (47). Briefly, for administration to female NCG mice (6–8 weeks of age; Shanghai Model Organisms Center, Inc.), virus- and Mycoplasma-free DLBCL patient 1 tissue were passaged *in vivo* subcutaneous (s.c). implantation into the shaved flanks of mice (n = 8 mice/group). Mice were treated with vehicle (5% v/v DMSO, 95% v/v HKI, i.p., QD), NSC632839 (20 mg/kg, 5% v/v DMSO, 95% v/v HKI, i.p., QD), for the indicated times. The development and progression of implanted tumors were monitored by digital caliper measurements of tumor length and width taken every 2 days for the duration of the experiment (13 days post-injection). Mice were sacrificed on day 16 of treatment. Tumor growth inhibition (TGI) rates were thus determined for both groups using the formula: TGI=((MTVcontrol-MTVtreated)/MTVcontrol) × 100.

All animal studies were performed in specific-pathogen-free, Helicobacter-free facilities in the Tongji University and the Chinese academy of Sciences Animal Resource Center following national, state and institutional guidelines and according to protocols approved by the Institutional Animal Care and Use Committee of both the Tongji University and the Chinese academy of Sciences.

#### CUT&RUN assay

CUT&RUN assay were carried out according to the manufacturer’s instructions (Hyperactive pG-MNase CUT&RUN Assay Kit for PCR/qPCR, Vazyme, HD101), briefly, 1 × 10^5^ A549 cells were treated with 5 uM NSC632839 in 24-well plates for 24 h, and then the cells were harvested. Cells were incubated with TP53 (Proteintech, 10442-1-AP) or rabbit IgG Isotype control (Proteintech, 30000-0-AP) for 2 h in RT, followed by MNase incubation at 4 °C for 1 h and fragmentation on ice for 90 min in the presence of Ca2^+^. At the same time, 1 pg of Spicke in DNA was added to each sample, then DNA fragments were released in water bath at 37 °C for 30 min, After DNA extraction, primer pairs targeting the TP53 binding site on DDR1-promoter were used. The enrichment level of TP53 on DDR1-promoter was detected by qPCR.

The sequences of the primer pair are as follows:

Forward 1 GGTTGCTTTTATCAGTGGGCCTG.

Reverse 1 CTCCAGCCTGGGCAACAGAG.

### Statistical analyses

Data are presented as mean ± SD or SEM as indicated. Unpaired *t* test, one-way ANOVA, two-way ANOVA and Wilcoxon test were used to determine statistically significant differences (∗*p* < 0.05, ∗∗*p* < 0.01, ∗∗∗*p* < 0.001, ∗∗∗∗*p* < 0.0001) using GraphPad Prism software.

## Ethics approval and consent to participate

Prior to acquisition of DLBCL primary patient samples, the subjects provided their informed consent to participate. All samples were obtained under the approval of the Tongji Hospital Review Board and our studies were performed in accordance with the Declaration of Helsinki. The patient’s information was list on Table 1, Table 2, Table 3.

## Data availability

For original data, please contact 2231495@tongji.edu.cn or jingy@tongji.edu.cn.

## Supporting information

This article contains supporting information.

## Conflict of interest

The authors declare that they do not have any conflicts of interest with the content of this article.

## Supporting information

Supplementary Figure 10

**Abbreviations**
cCol-I, cleaved collagen I
CHX, cycloheximide
DDR1, Discoidin Domain Receptor 1
DLBCL, diffuse large B-cell lymphoma
DUBs, deubiquitylating enzymes
ECM, extracellular matrix
FISH, Fluorescence in situ hybridization
LUAD, Lung Adenocarcinoma
LUSC, Lung Squamous Cell Carcinoma
NSCLC, non-small cell lung cancer
TMA, tissue microarray
