# Supplementary material for: Ubiquitin-specific protease 7-mediated stabilization of discoidin domain receptor 1 drives progression of TP53-Mutant cancers
Source: J Biol Chem. 2025 Jul 24;301(9):110515. doi: 10.1016/j.jbc.2025.110515 (PMC12390941; doi:10.1016/j.jbc.2025.110515)
Supplement: Supplementary information [file mmc1.docx]

**Supplementary Figure legends:**

**Supplementary Figure 1. DDR1 is overexpressed and serves as a poor prognostic marker in lung cancer and DLBCL.** (A) DDR1 expression differences between tumor and non-tumor tissues in LUAD and LUSC were analyzed using the "Expression Analysis" module of Gene Expression Profiling Interactive Analysis version 2 (GEPIA2) (<http://gepia2.cancer-pku.cn/>). (B) Comparison of DDR1 gene expression levels between DLBCL patients and normal controls. Boxplots represent DDR1 expression levels in tumor samples from TCGA-DLBC patients (red) and normal tissues from GTEx (blue). Statistical significance was assessed using the t-test (p-values are indicated). (C) Kaplan-Meier plots for overall survival (OS) and first progression survival (FP) in lung cancer were generated using Kaplan-Meier Plotter (<https://kmplot.com/analysis/>).

**Supplementary Figure 2. ABPP-DUBome, mass spectrometry analysis, and co-expression studies identified USP7 as a deubiquitinase that stabilizes DDR1.** (A) Schematic strategy for the activity-based DUB profiling assay (ABPP) of NSC632839 using recombinant DUB proteins and ubiquitin-based activity probes (Ub-AMC) (up panel). DUB profiling studies: USP1, USP7, USP11, USP12, USP46 and USP47 were identified as potent and unique targets of NSC632839 (down panel). (B) Mass spectrometry identified USP7 and USP10 as potential DUBs that stabilize DDR1. (C) HEK293T cells were co-transfected with vectors encoding USP2, USP7, USP11 or USP12 and DDR1 to evaluate the stabilizing effect of each DUB on DDR1. DUBs were detected using either HA- or MYC-tag-specific antibodies. Shown are the representative results of three independent experiments.

**Supplementary Figure 3. USP7 is an adverse prognostic marker in lymphoma.** Survival analysis based on USP7 gene expression in lymphoma patients. Kaplan-Meier survival curves depict the overall survival of patients stratified by high vs. low USP7 expression levels. Data were obtained from multiple lymphoma datasets (GSE10846, GSE53786, GSE181063) and analyzed using the survival and survminer packages in R. Statistical significance was assessed using the log-rank test, with p-values indicated on the plot.

**Supplementary Figure 4. Selective USP7 inhibition induces DDR1 degradation without additive effects of NSC632839.**  (A) The enzymatic activity assays with purified USP7 protein were conducted to confirm the direct inhibition of USP7 by NSC632839 at biologically relevant concentrations. (B) A549 cells were treated with the USP7 selective inhibitor FT671 for 24 h, followed by cell lysis to detect protein levels of DDR1, TP53 and β-Actin. (C) A549 cells were treated with NSC632839, with or without the USP7-selective inhibitor FT671, for 24 hours, followed by cell lysis and immunoblotting to assess DDR1 and GAPDH protein levels. (D) The effect of NSC632839, with or without FT671 (5 μM), on A549 cell proliferation was evaluated using the CTG assay. (E-F) USP7 gene knockdown was performed in A549 cells, followed by treatment with or without NSC632839. The growth curves of the different cell groups were assessed using the CTG assay, with knockdown efficiency shown in (F). Mean ± SD; Two-way ANOVA. Data shown are representative of three independent experiments. Mean ± SD. *P < 0.05, **P < 0.01, ***P < 0.001, ****P < 0.0001.

**Supplementary Figure 5. NSC632839 inhibits cell growth in a DDR1-dependent manner.** (A) A549 cells were transduced with a lentiviral vector encoding DDR1 to achieve overexpression. DDR1 and GAPDH protein levels were assessed by immunoblotting. (B) Cells were generated as described in (A), and the growth curves of different cell groups were assessed using the CTG assay. Mean ± SD; Two-way ANOVA. (C) A549 SCR and shUSP7 monoclonal cell lines were established as described. To achieve DDR1 overexpression, A549 shUSP7 monoclonal cells were further transduced with a lentiviral vector encoding DDR1. Protein levels of DDR1, USP7, and GAPDH were evaluated by immunoblotting. (D) Cells were generated as described in (C), and the growth curves of different cell groups were assessed using the CTG assay. Mean ± SD; Two-way ANOVA. Shown are the representative results of three independent experiments. *P < 0.05, **P < 0.01, ***P < 0.001, ****P < 0.0001.

**Supplementary Figure 6. Treatment with NSC632839 in the A549-CDX model exhibited a significant anti-tumor effect with no apparent toxicity.** (A) Mice were sacrificed on day 15 of treatment, and photographs of the excised tumors were obtained. (B) Changes of body weight over time were monitored every day for the duration of the experiment (15 days post-injection) (n = 5/group). Initial body weight was set as 100%. Mean ± SD. (C) pAKT levels in tumor tissue were determined by western blotting, followed by quantification and representation in a bar graph. Mean ± SD; Unpaired t-test. *P < 0.05, **P < 0.01, ***P < 0.001, ****P < 0.0001.

**Supplementary Figure 7. DDR1 is highly expressed in TP53 mutant cell lines and DLBCL patient samples.** (A) Transcriptomic analysis: A total of 60 DLBCL patient samples were grouped into TP53-WT, TP53-MISS (deletion), and TP53-MUT categories. The expression levels of DDR1 mRNA were analyzed across these groups. Mean ± SD, Wilcoxon test. (B) USP7 mRNA expression: The relative mRNA levels of USP7 were measured in the TP53-WT, TP53-MISS, TP53-MUT, and TP53-ALL MUT groups using the 60 DLBCL patient samples. Mean ± SD; Unpaired t-test. *P < 0.05, **P < 0.01, ***P < 0.001, ****P < 0.0001.

**Supplementary Figure 8. NSC632839 potentiates TP53-mediated transcriptional repression of DDR1.** (A) The DDR1 promoter sequence is shown with TP53 binding sites indicated. The effect of TP53 binding site loss was assessed by introducing the depicted mutations. (B) A549 cells were collected and the recruitment change of TP53 on DDR1 promoter were confirmed by CUT & RUN assay. Mean ± SD (n = 8); Unpaired t-test. (C-D) HEK-293T cells were co-transfected with a DDR1 promoter-driven firefly luciferase reporter, with or without TP53, and treated with or without NSC632839 (5 μM) for 16 hours before harvesting for luciferase activity measurement, normalized to CTG. Mean ± SD (n = 6); Unpaired t-test for (C); One-way ANOVA for (D). (E) TP53 knockdown: TP53 was silenced in HEK-293T and A549 cells by transducing puromycin-resistant shRNA-1/2 constructs via lentiviral delivery. Western blotting was used to detect the protein levels of DDR1, p53 and GAPDH. Shown are the representative results of three independent experiments. *P < 0.05, **P < 0.01, ***P < 0.001, ****P < 0.0001.

**Supplementary Figure 9. Selective inhibition of USP7 induces the cytotoxicity of TP53-mutant DDR1-positive PDCs *ex vivo*.** Patient-derived cells (PDCs) from DLBCL patients (n = 3) were treated with the USP7 selective inhibitor FT671 at varying concentrations for 48 h. Cell proliferation was assessed using the CellTiter-Glo® (CTG) assay. Mean ± SD. Shown are the representative results of three independent experiments.

**Supplementary Figure 10. Treatment with NSC632839 in PDX model demonstrated no significant toxicity.** (A) Changes of body weight over time were monitored every day for the duration of the experiment (16 days post-injection) (n = 8/group). Initial body weight was set as 100%. Mean ± SD. (B) H&E staining of the liver, kidney, and spleen from experimental mice showed no evident toxicity of NSC632839. Scale bar represents 50-100 μm.
