# Supplementary figures and images for: Ubiquitin-specific protease 7-mediated stabilization of discoidin domain receptor 1 drives progression of TP53-Mutant cancers
Source: J Biol Chem. 2025 Jul 24;301(9):110515. doi: 10.1016/j.jbc.2025.110515 (PMC12390941; doi:10.1016/j.jbc.2025.110515)

Supplementary Figure 1

A

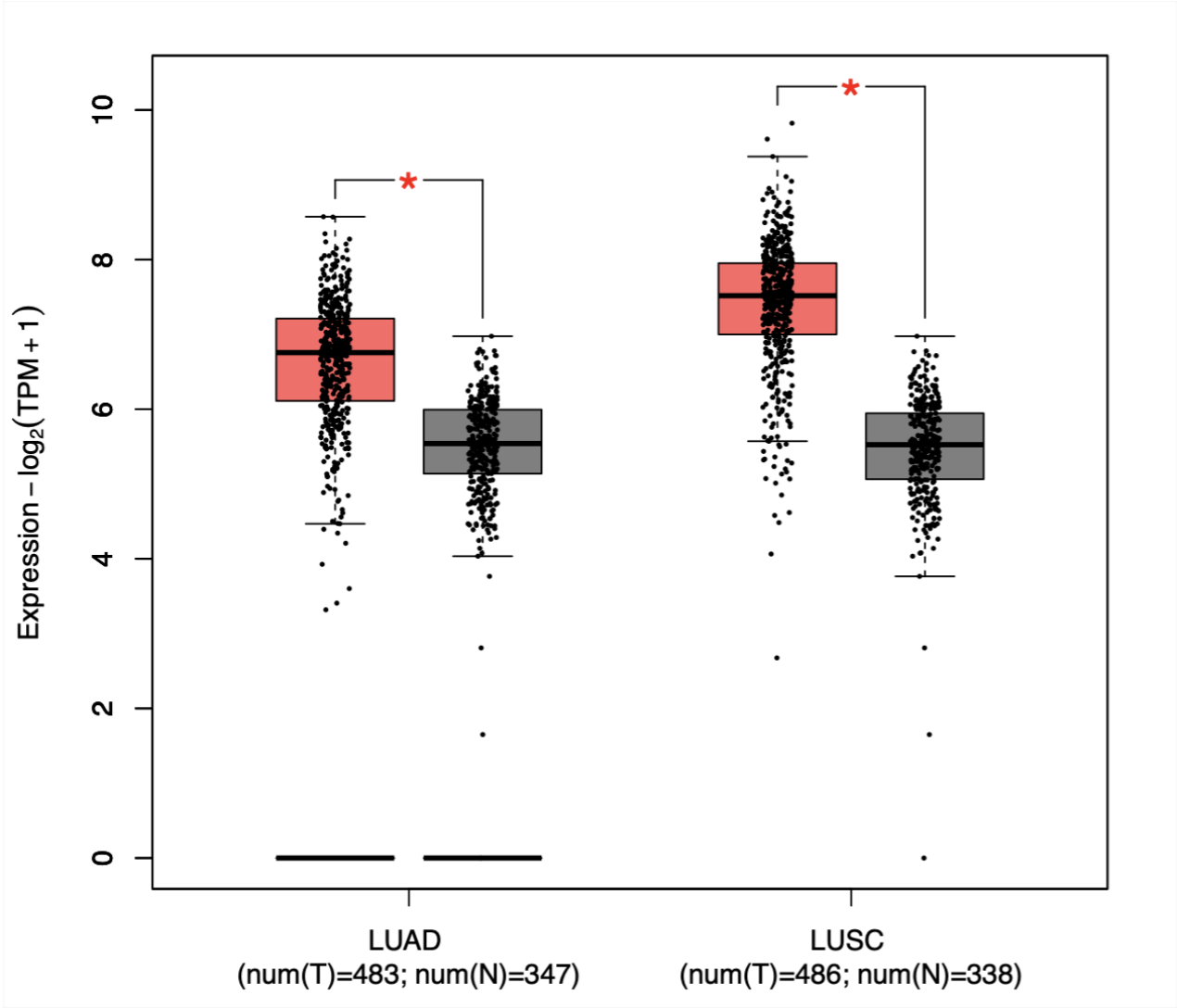

C

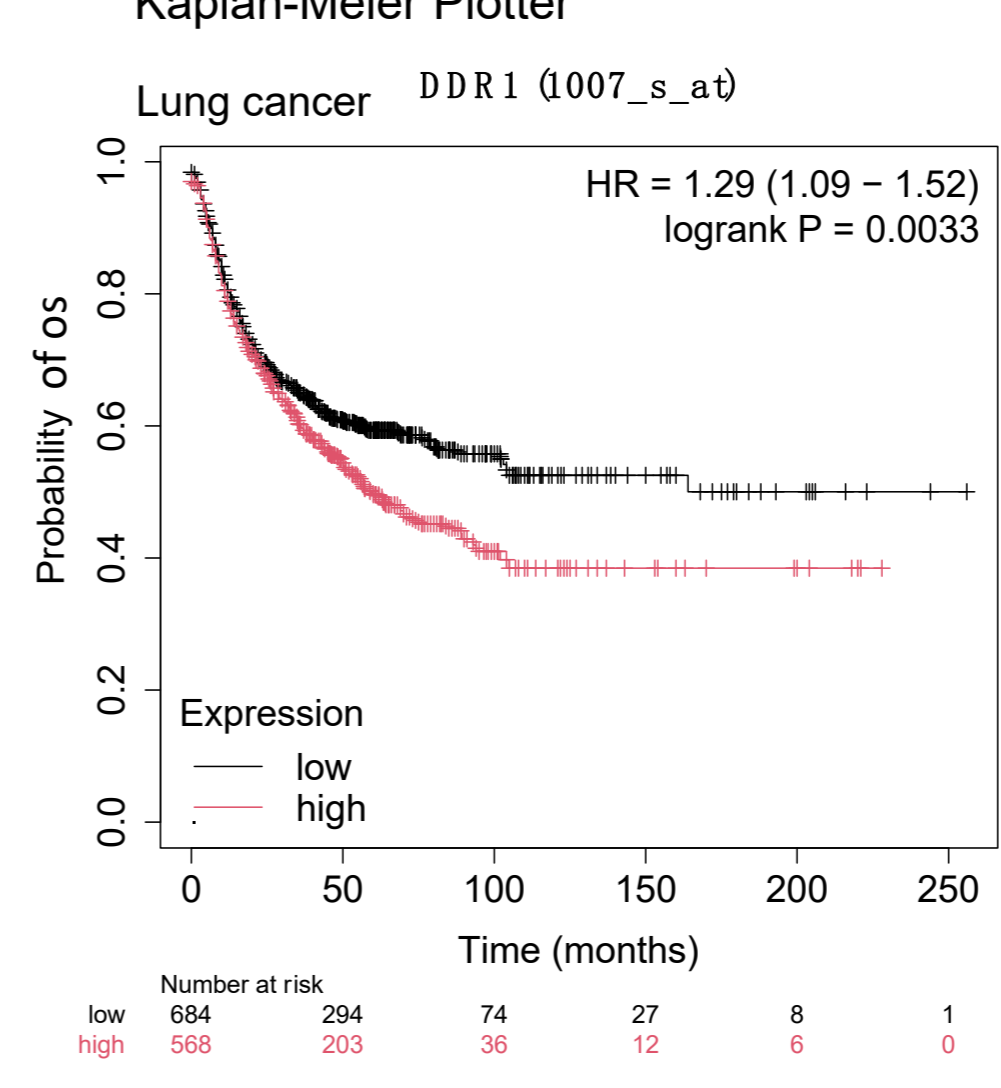

B

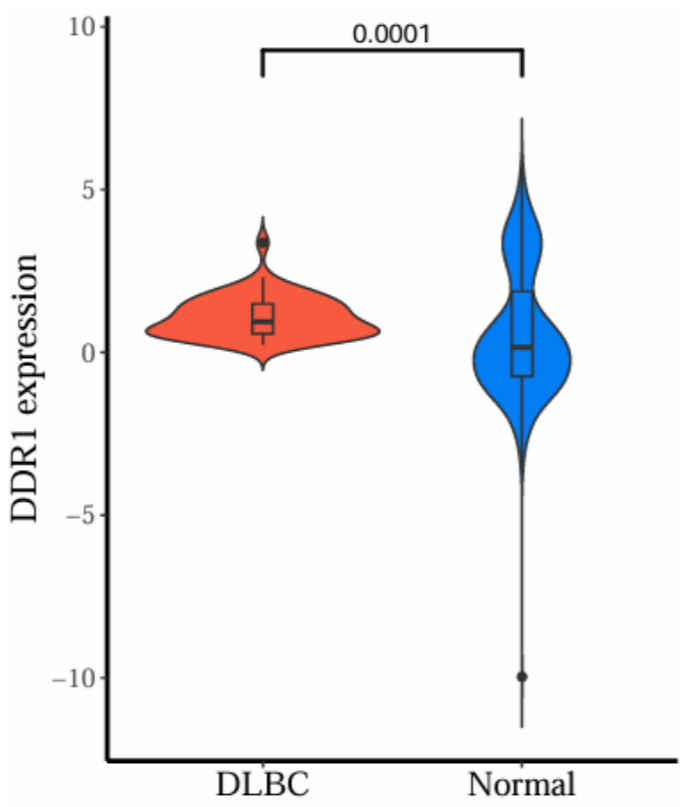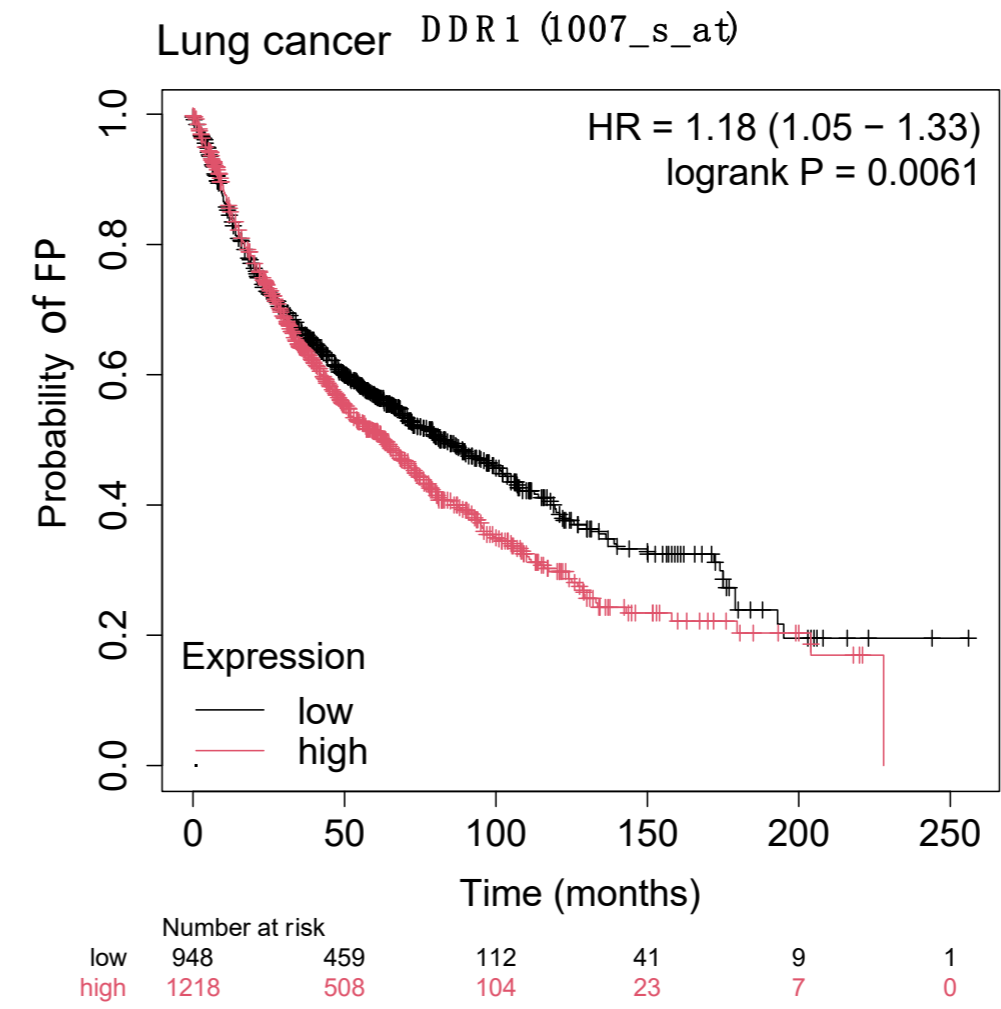

Supplement: Supplementary Figure 1 [file mmc2.pdf]

# Supplementary Figure 3

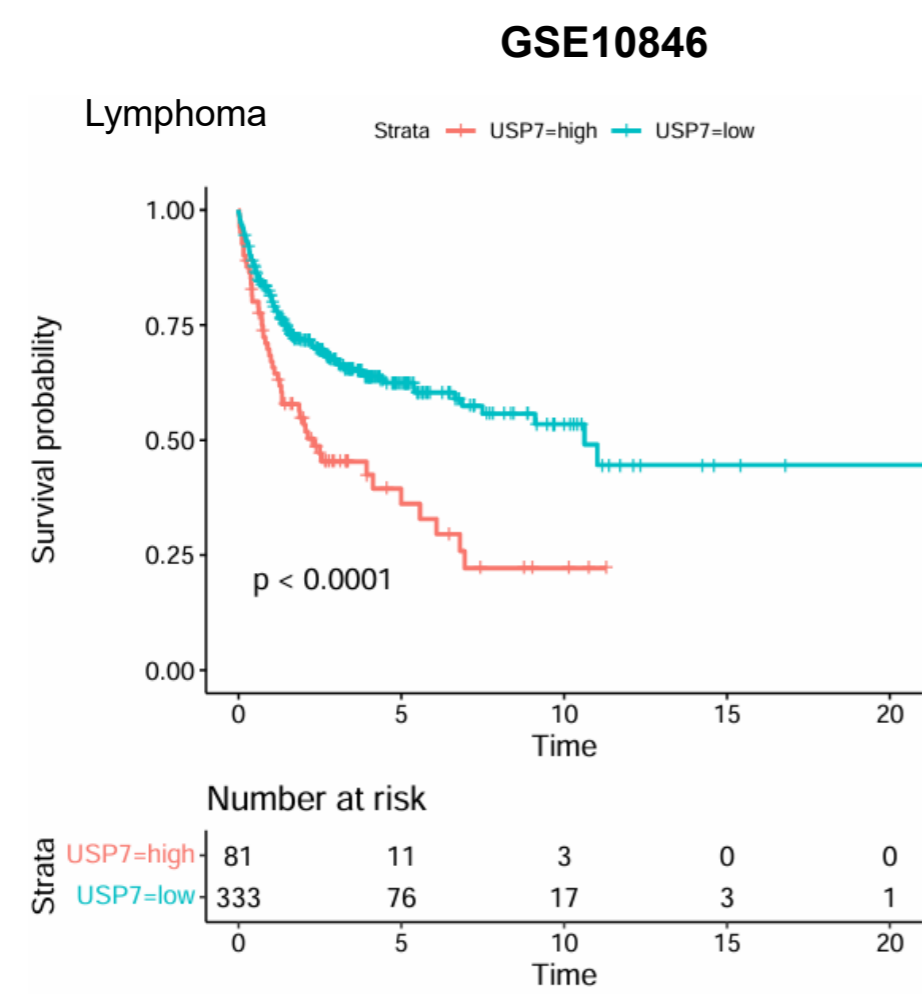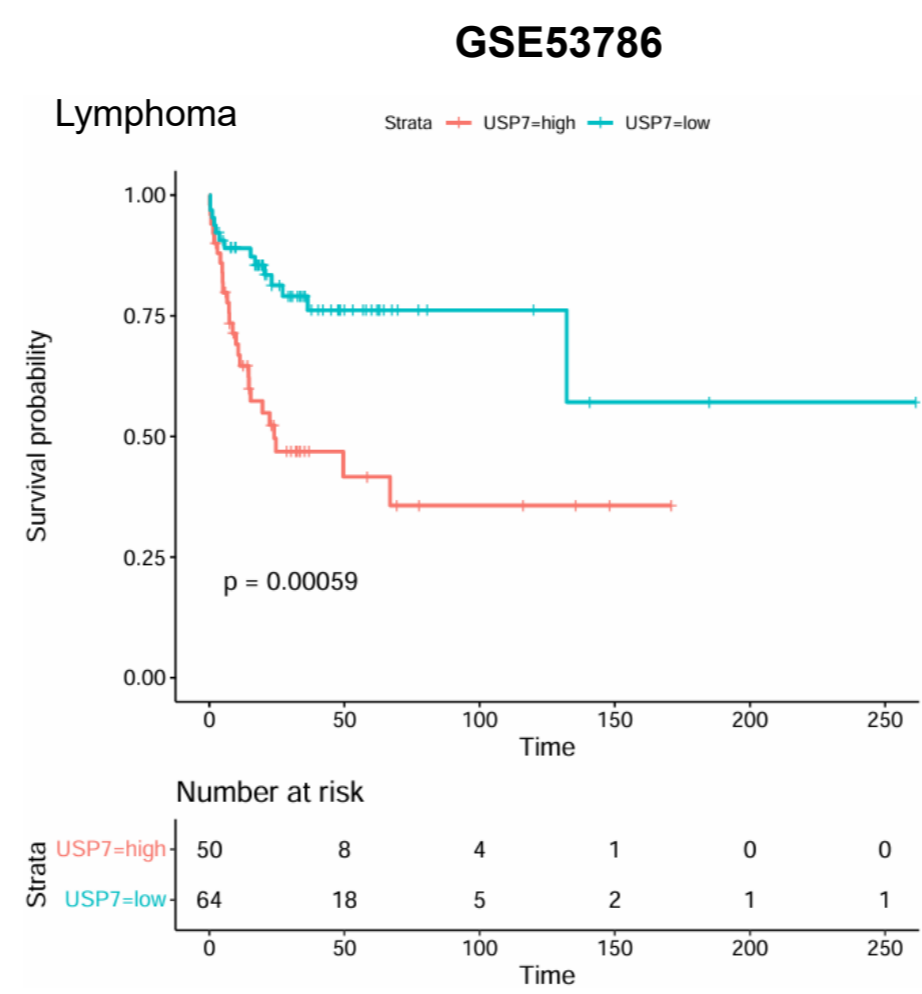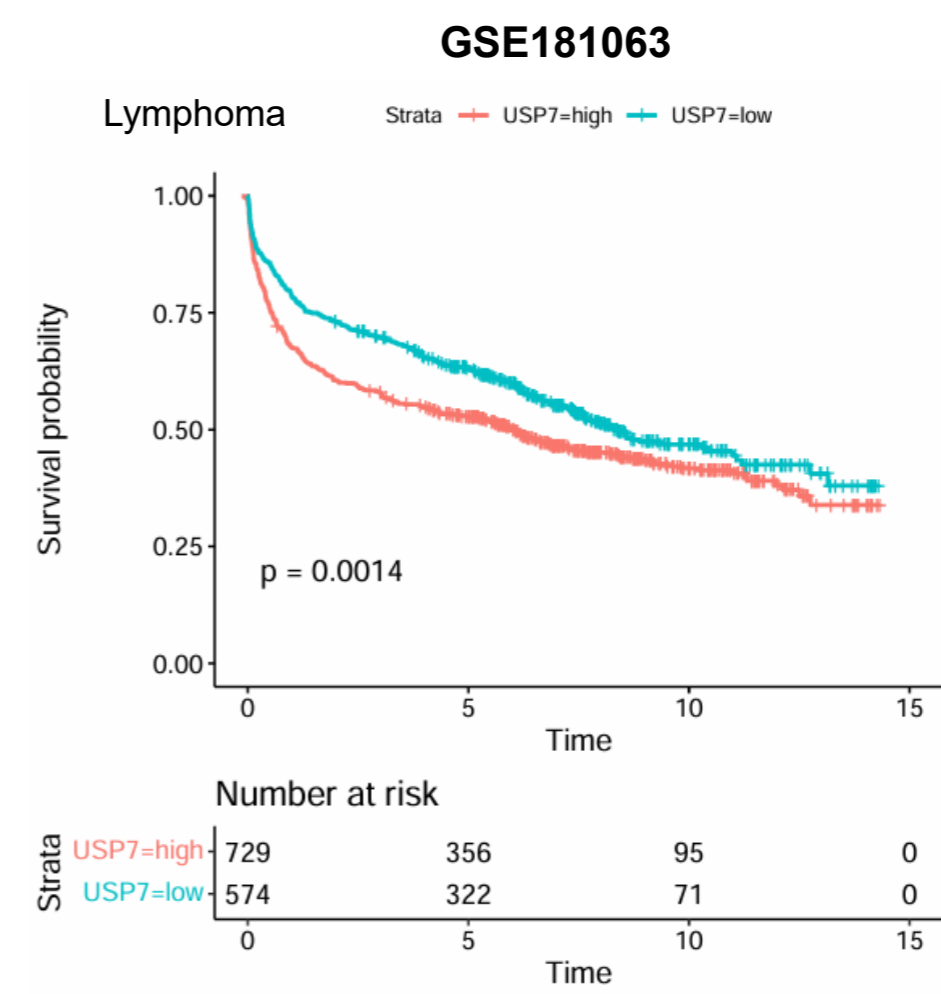

Supplement: Supplementary Figure 3 [file mmc4.pdf]

Supplementary Figure 4

A

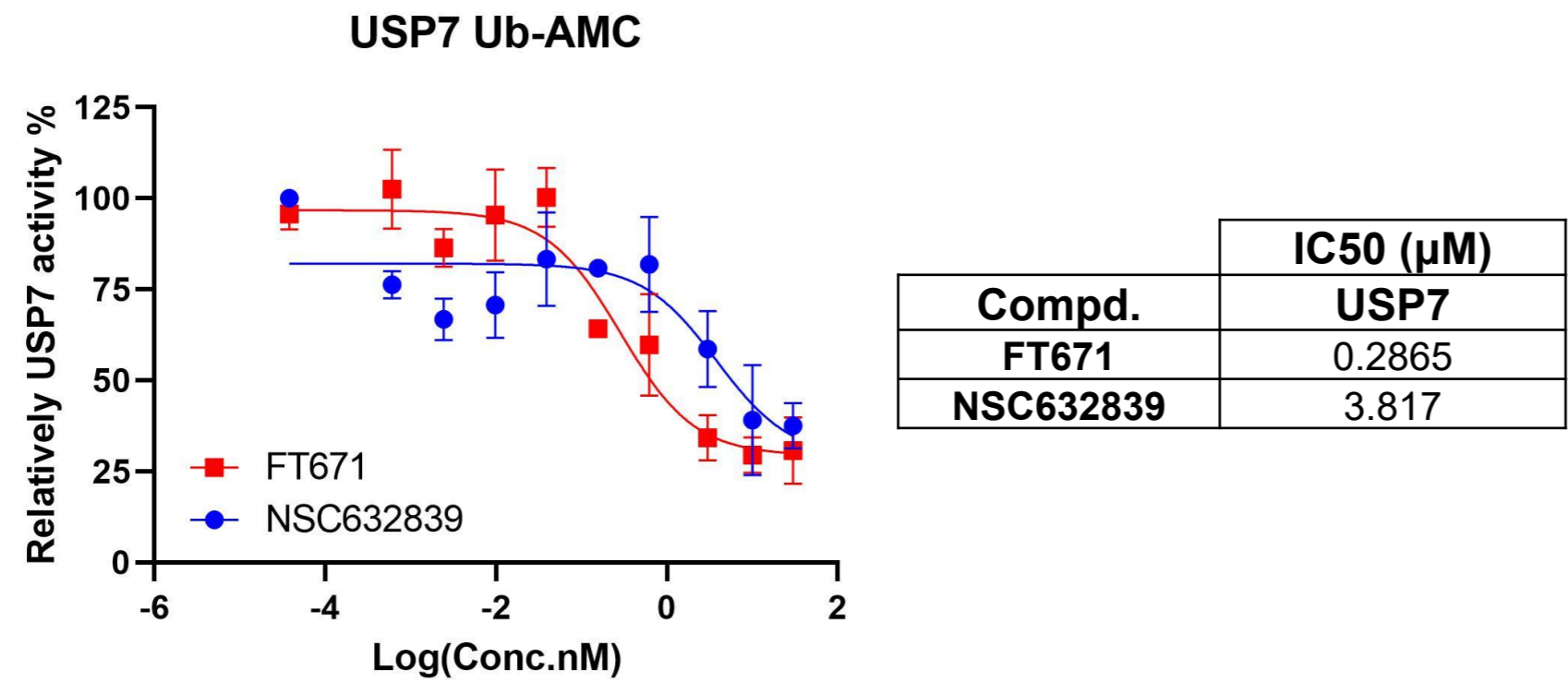

B

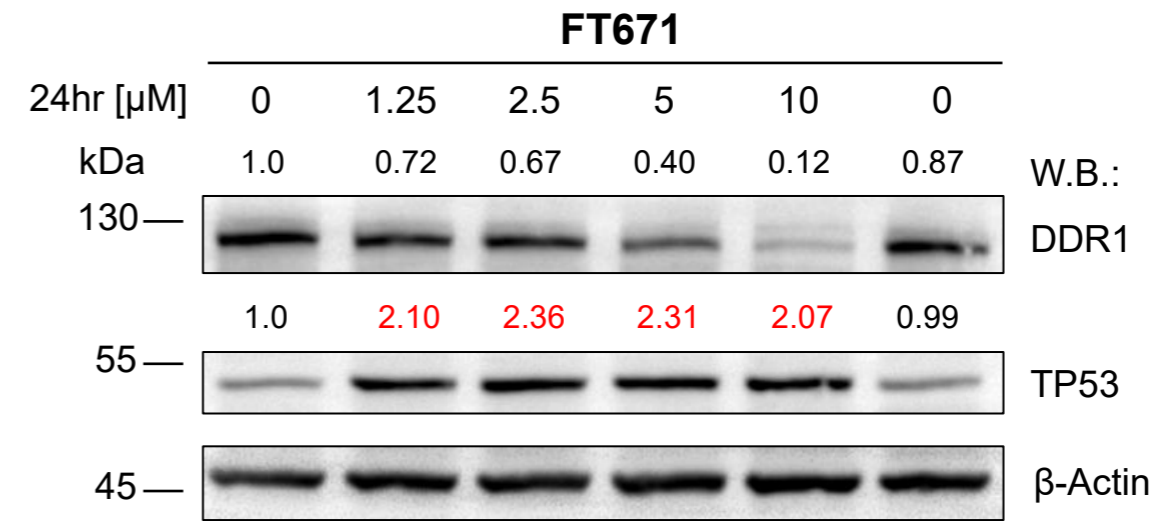

C

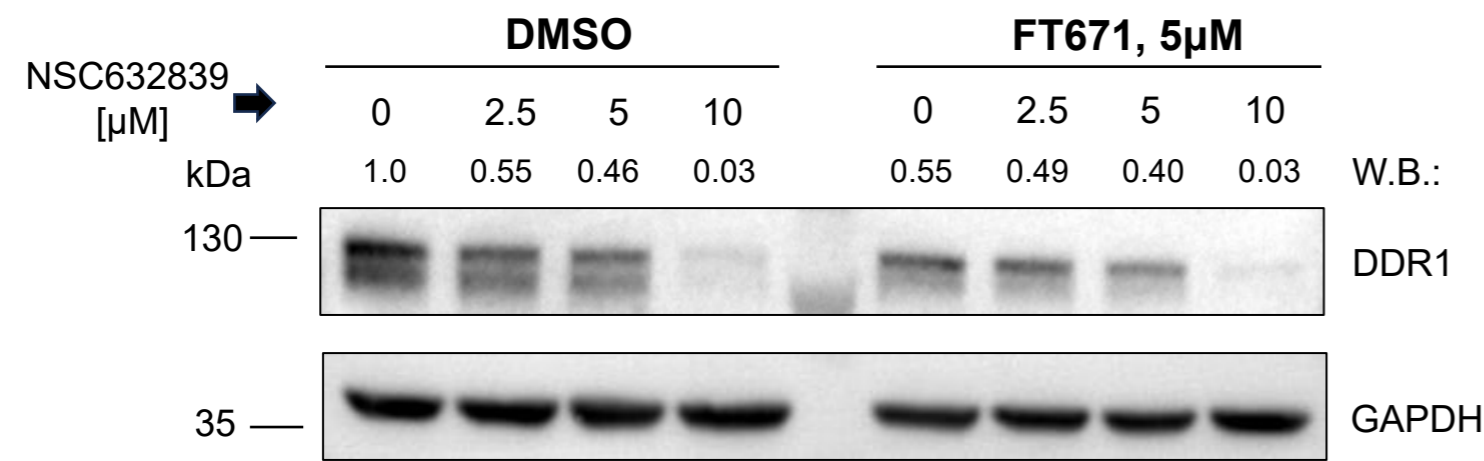

D

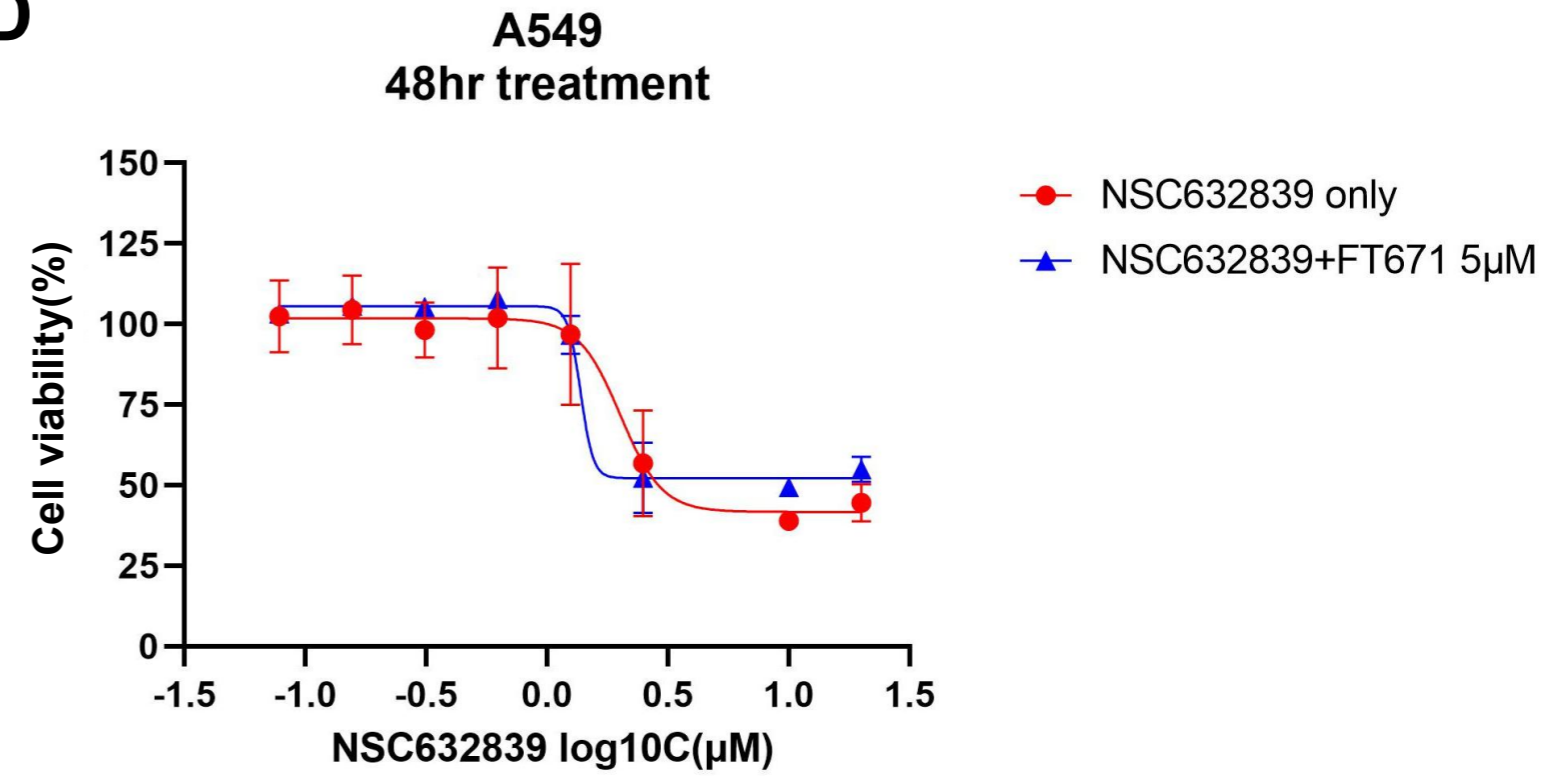

E

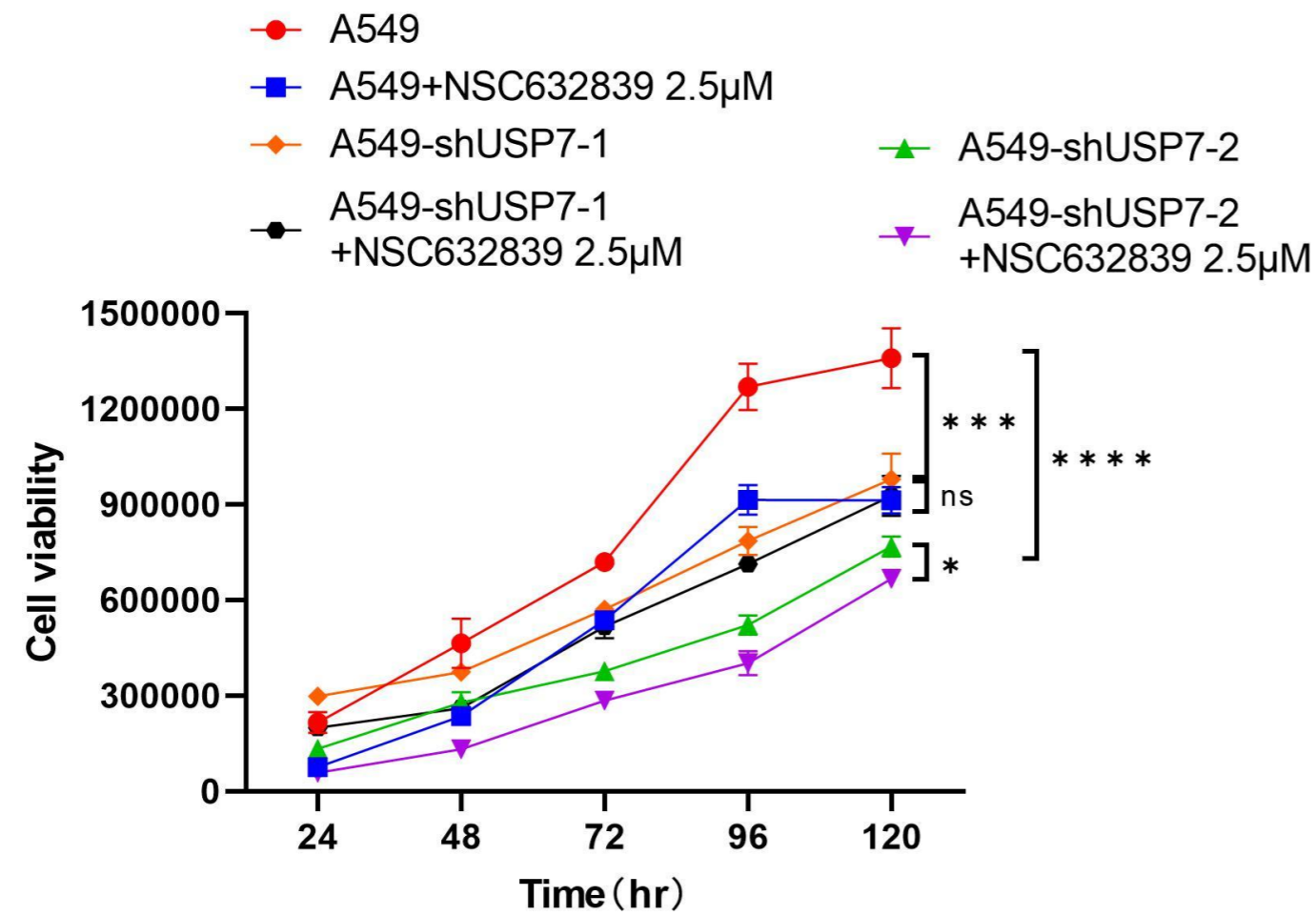

F

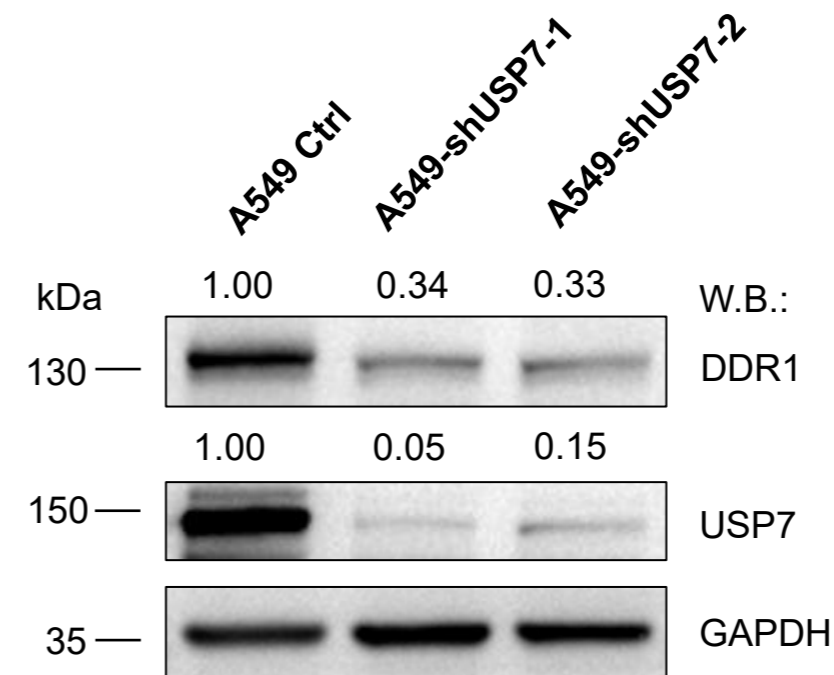

Supplement: Supplementary Figure 4 [file mmc5.pdf]

## Supplementary Figure 5

**A**

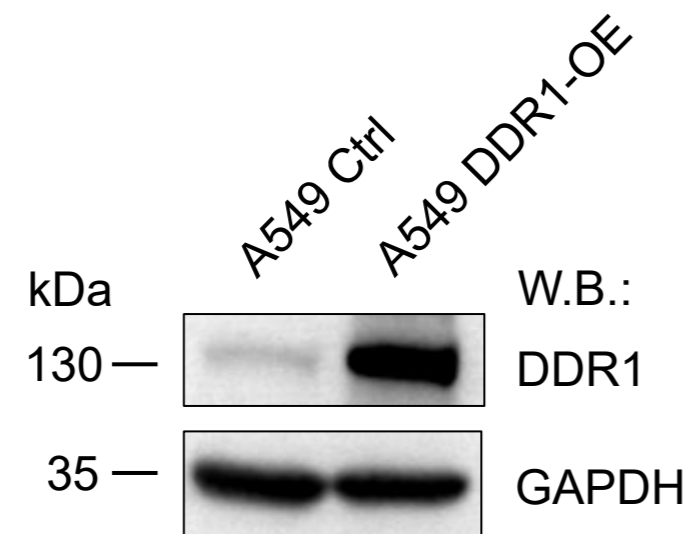

**B**

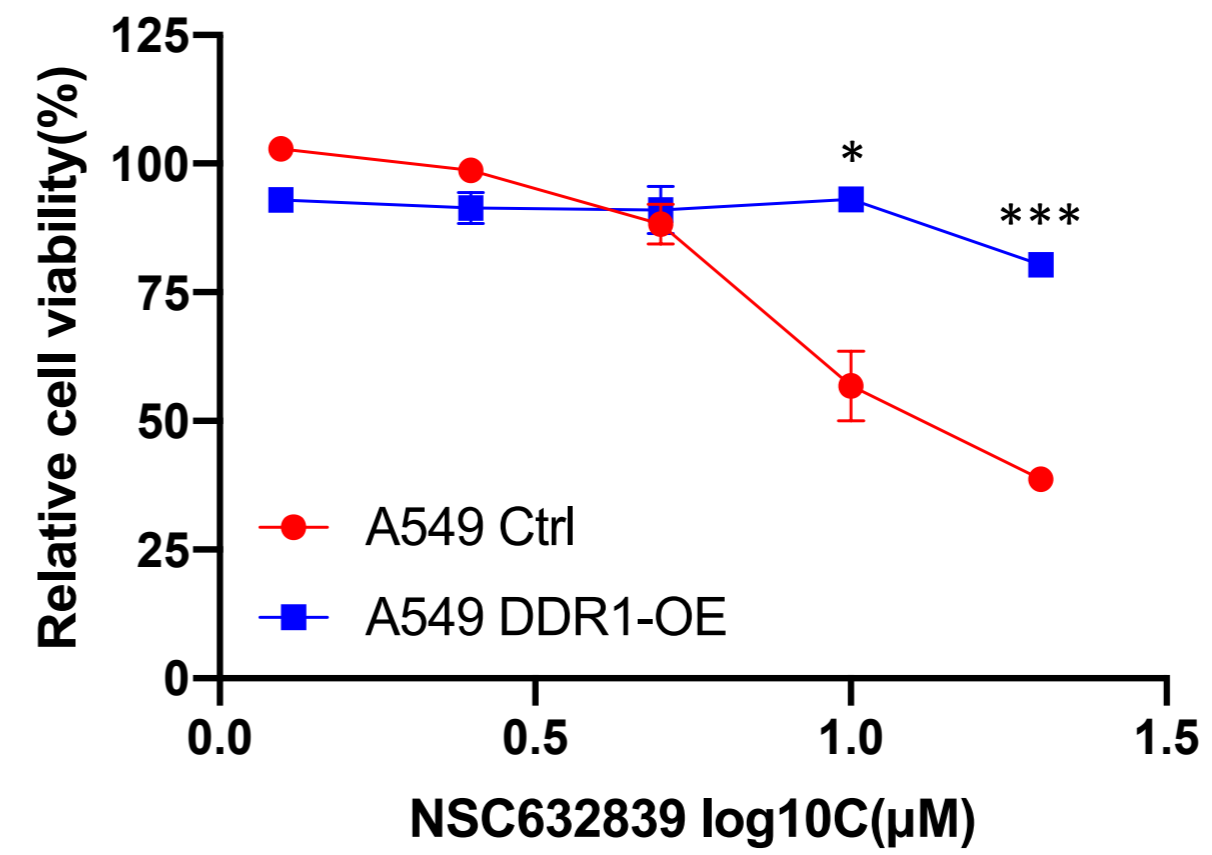

**C**

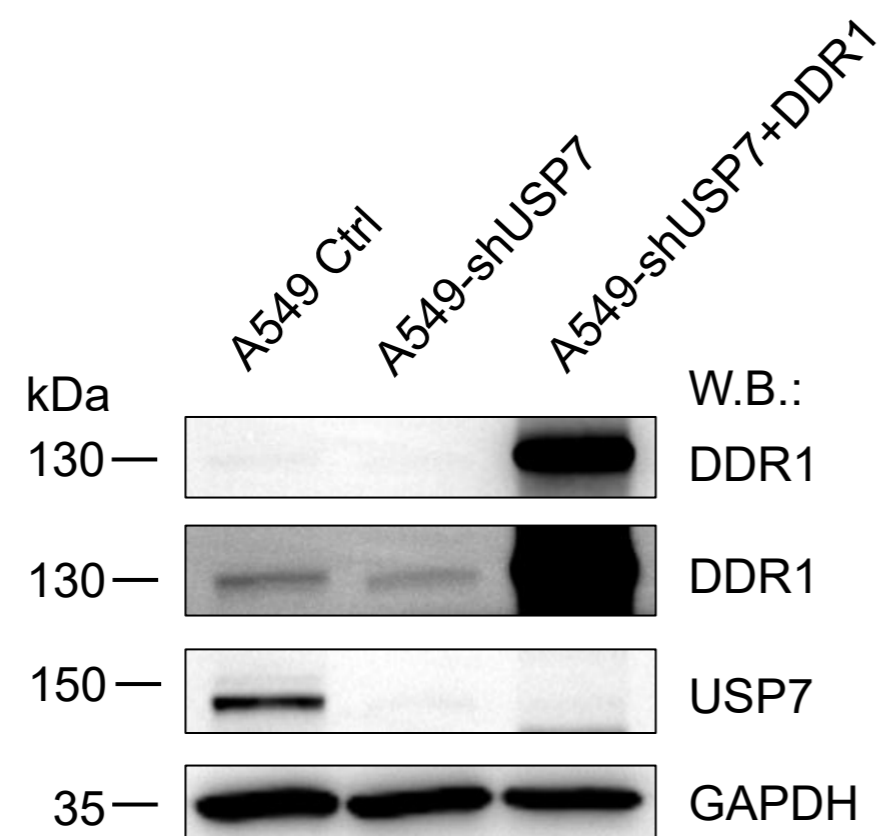

**D**

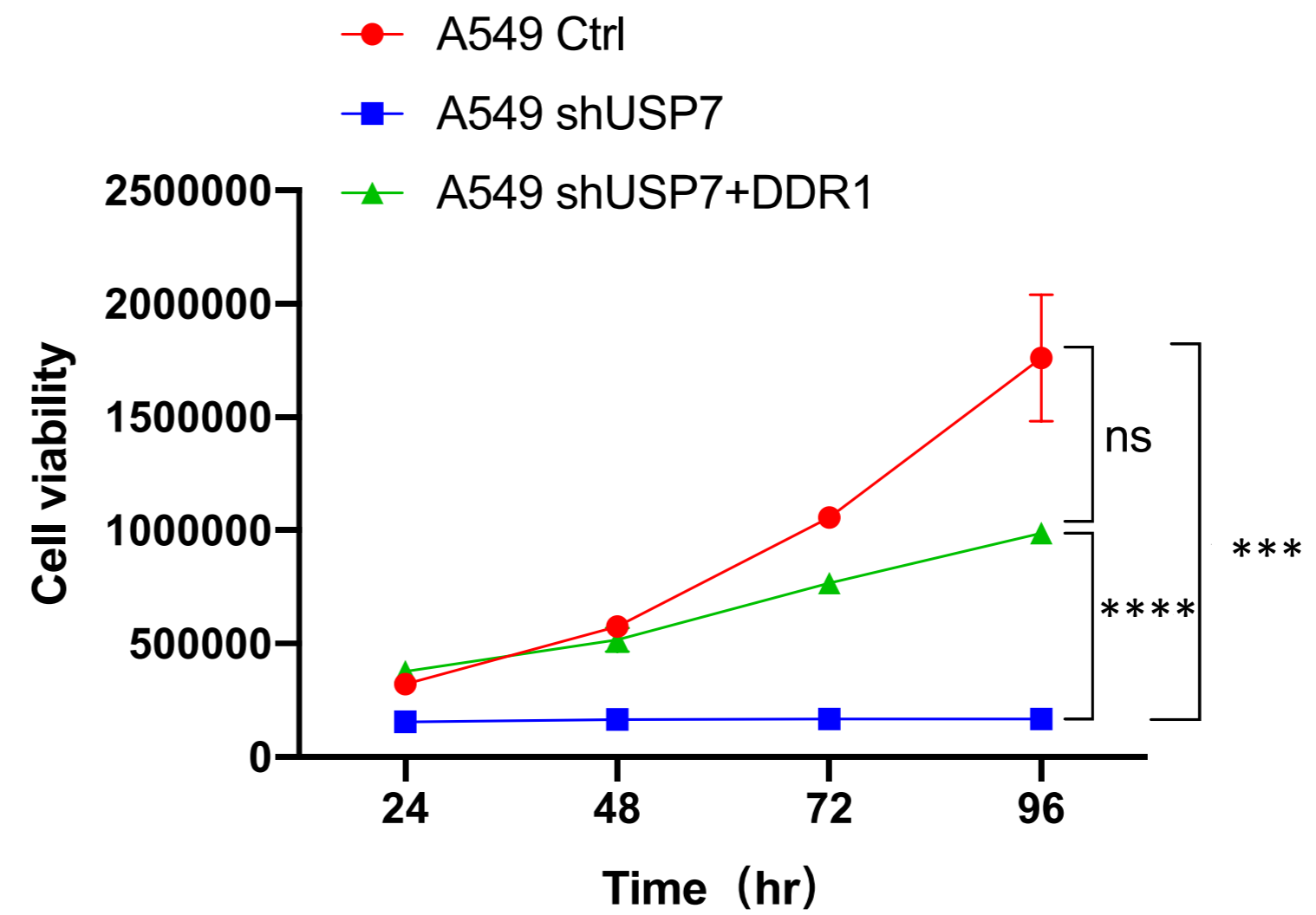

Supplement: Supplementary Figure 5 [file mmc6.pdf]

Supplementary Figure 6

A

A549

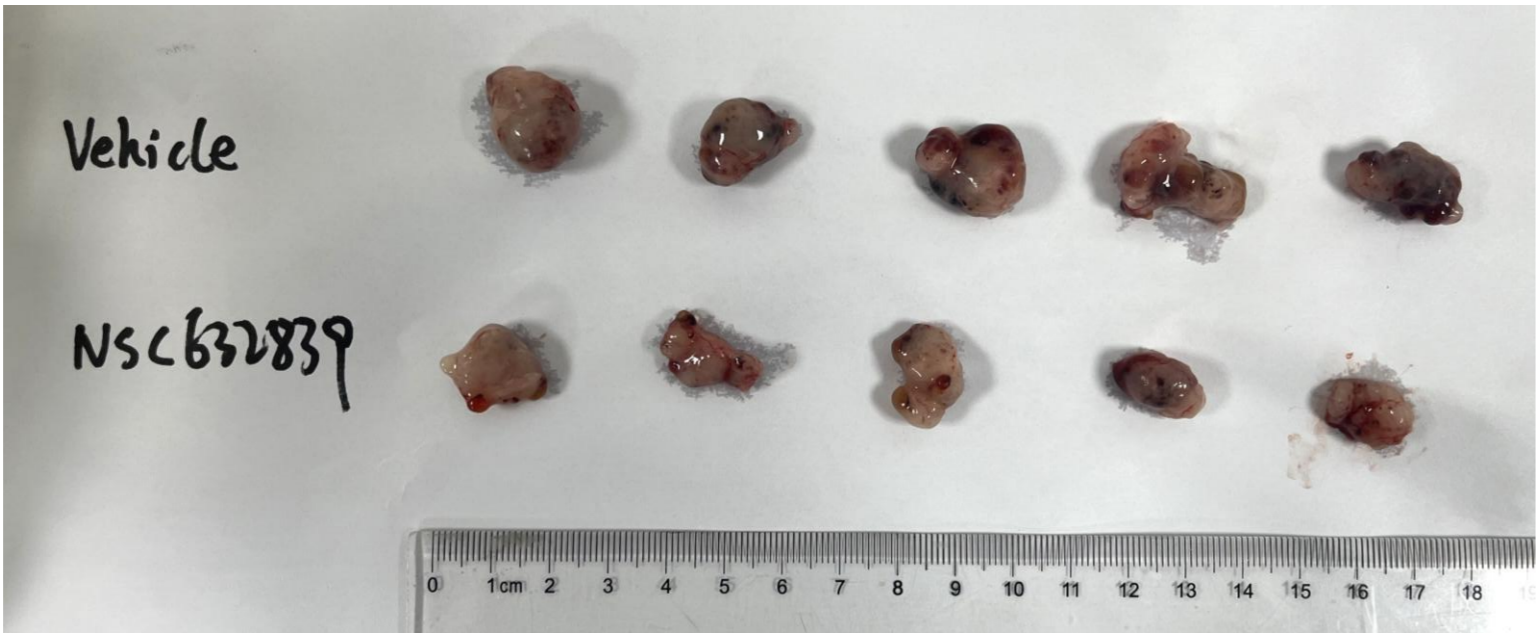

B

A549  
Body Weight

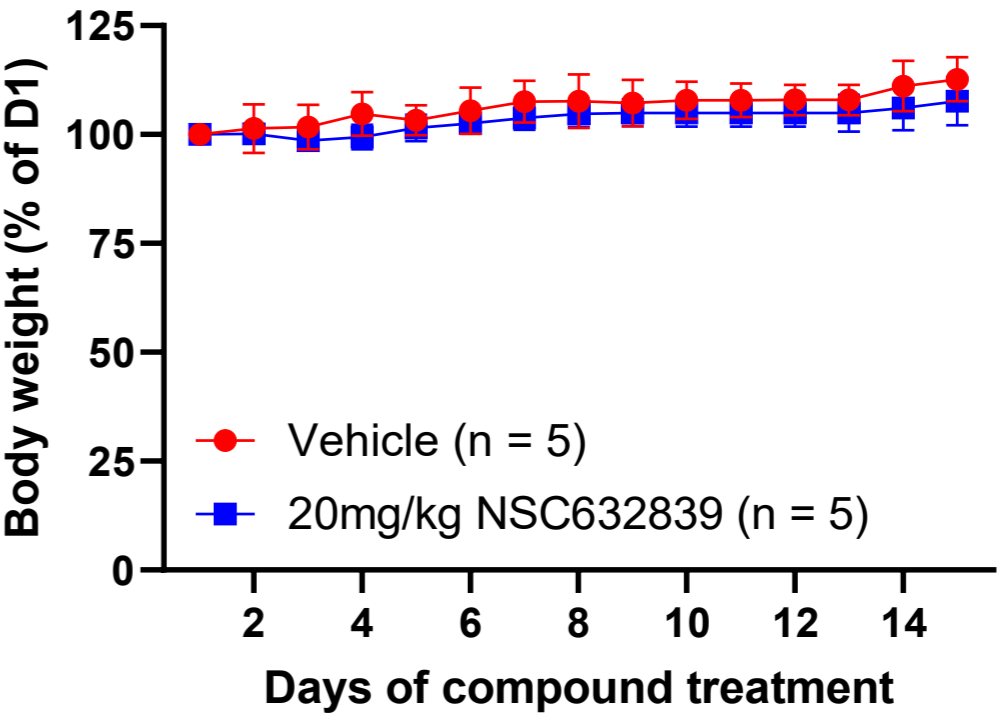

C

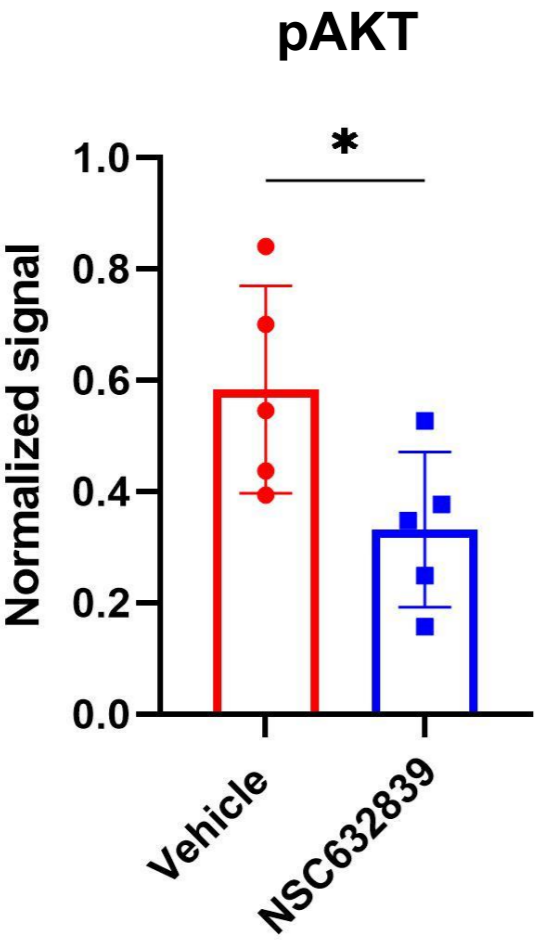

Supplement: Supplementary Figure 6 [file mmc7.pdf]

Supplementary Figure 7

**A**

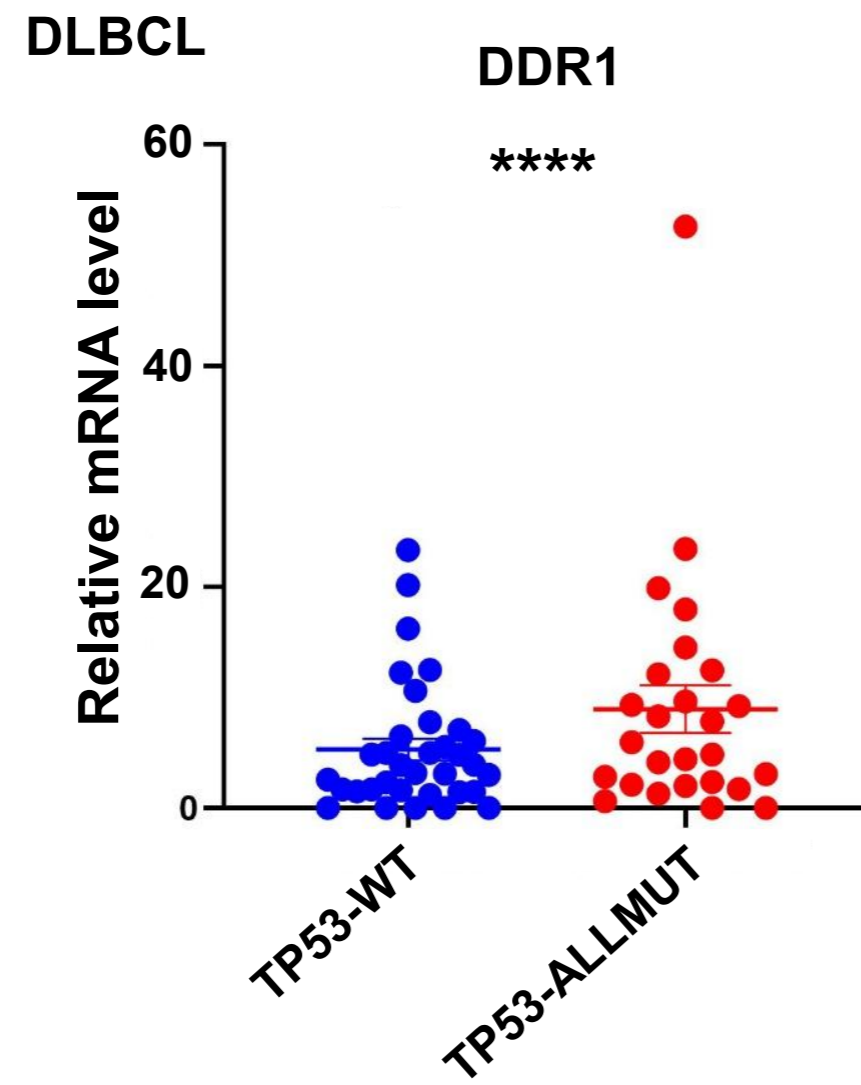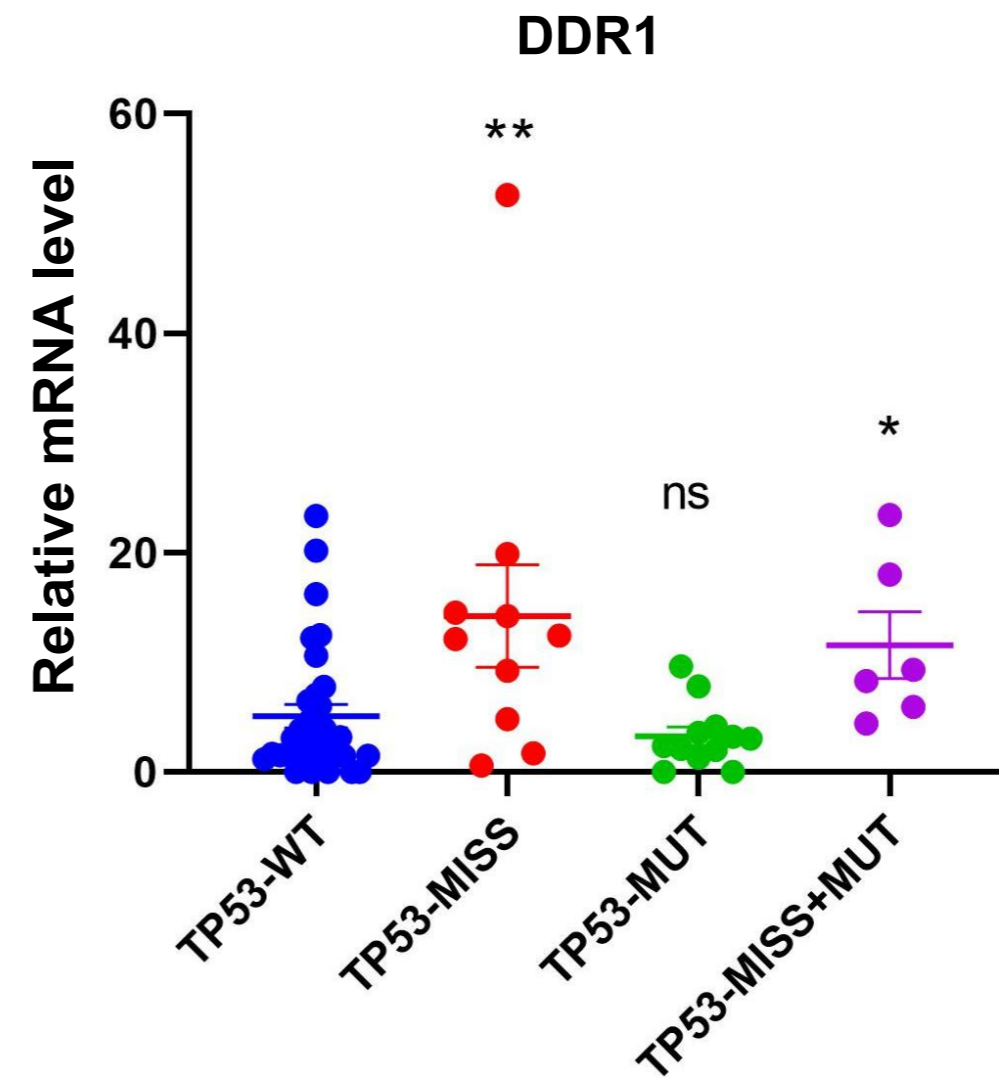

**B**

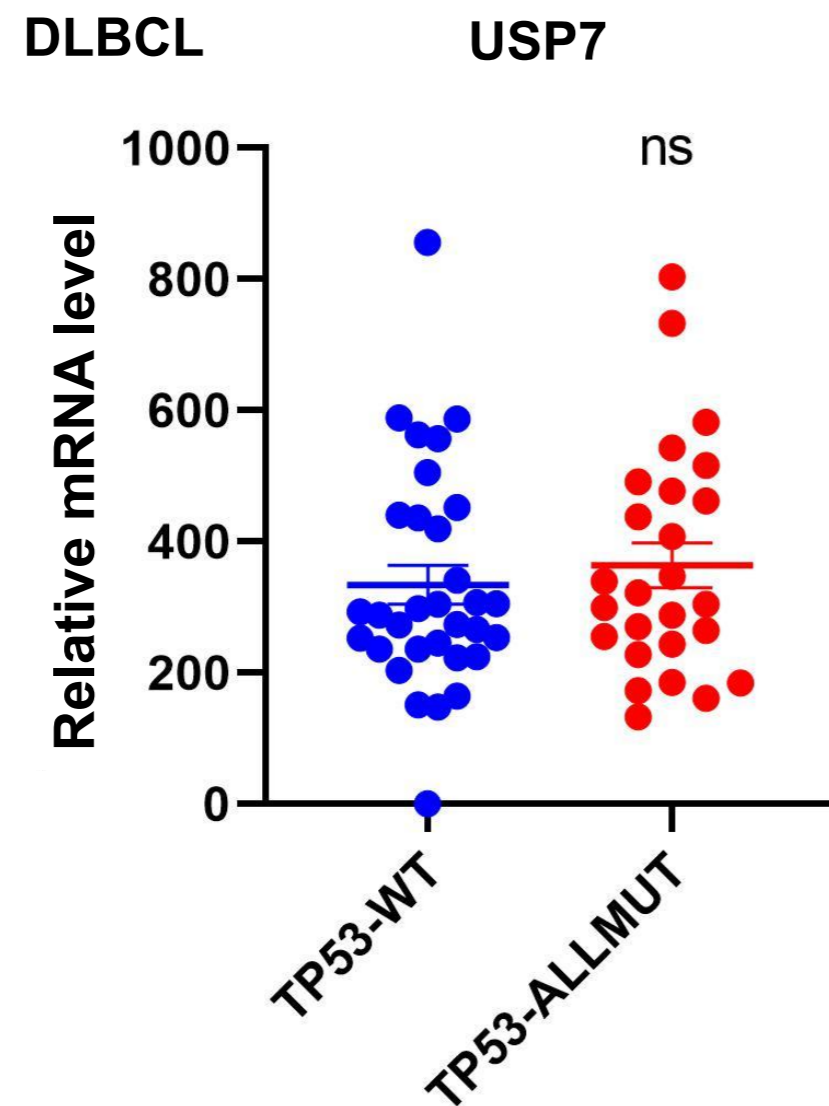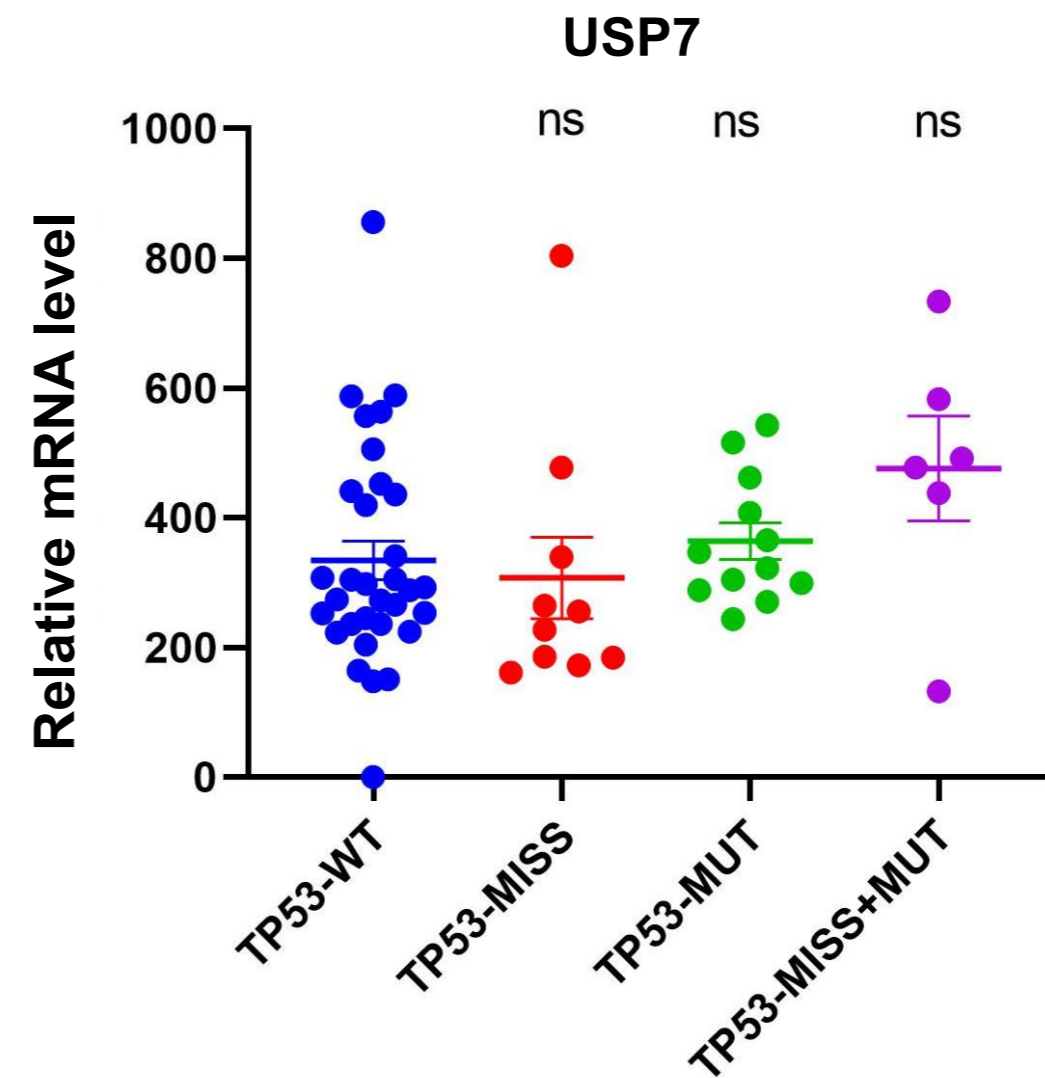

Supplement: Supplementary Figure 7 [file mmc8.pdf]

Supplementary Figure 8

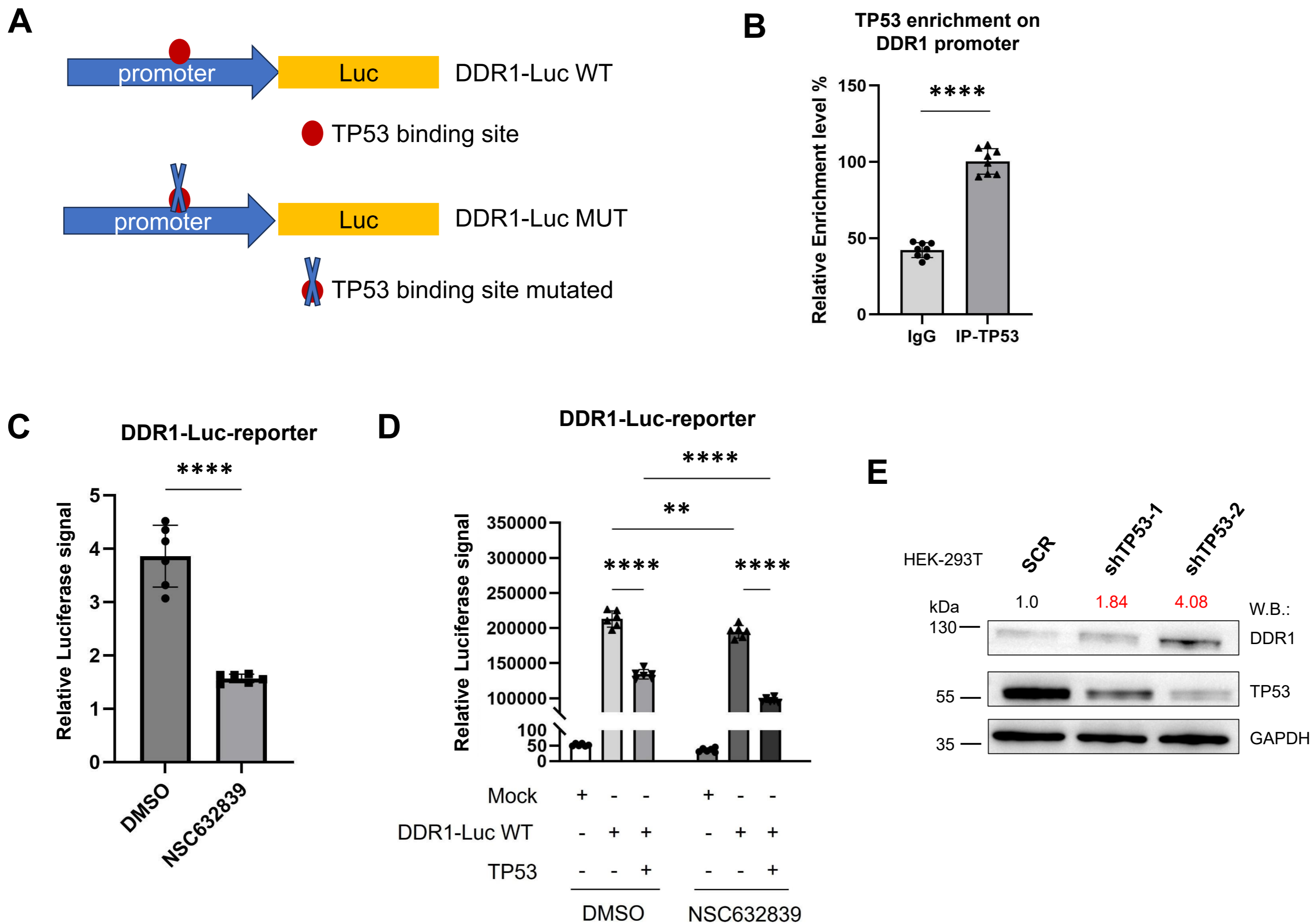

Supplement: Supplementary Figure 8 [file mmc9.pdf]

Supplementary Figure 9

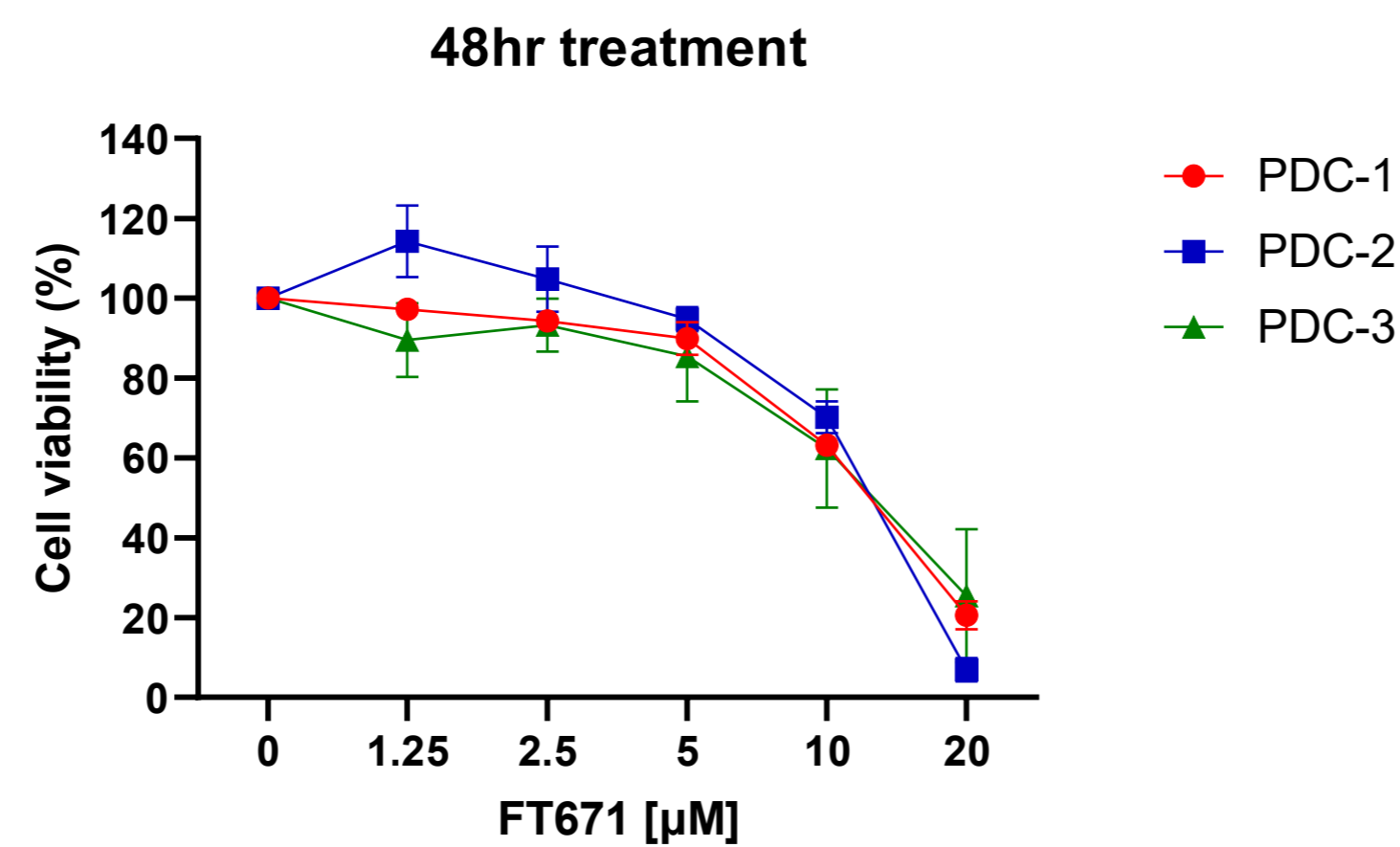

Supplement: Supplementary Figure 9 [file mmc10.pdf]

Supplementary Figure 10

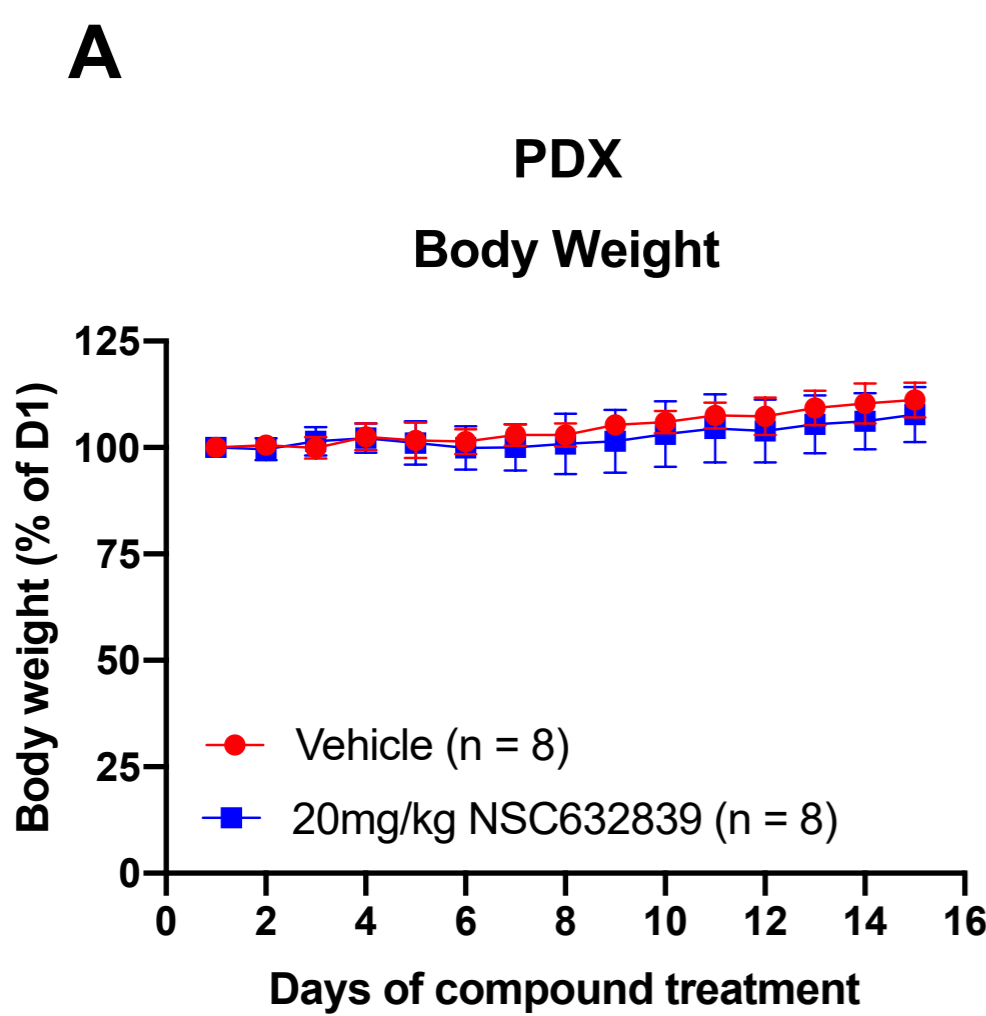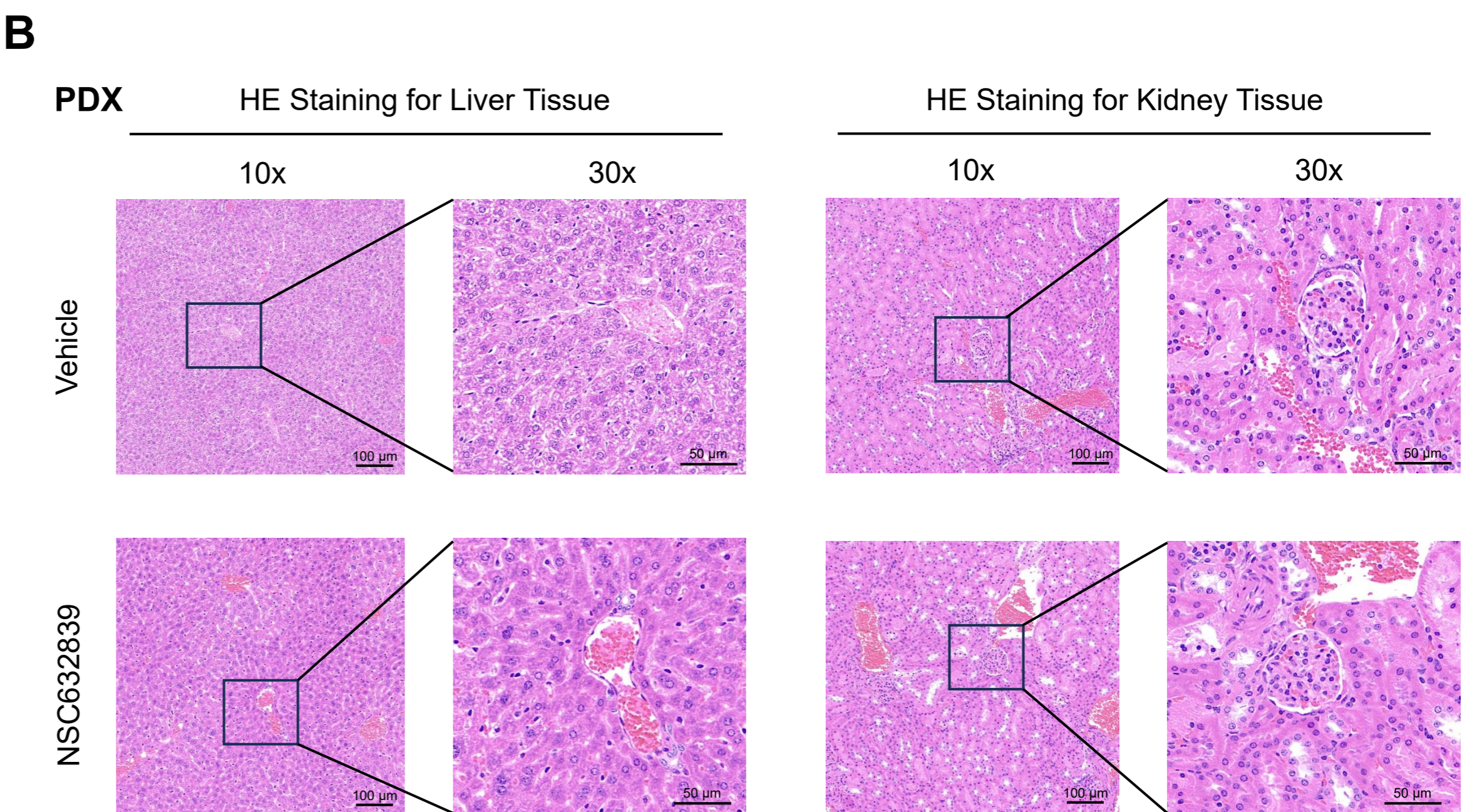

Supplement: Supplementary Figure 10 [file mmc11.pdf]
