# Supplementary material for: Ubiquitin-specific protease 7-mediated stabilization of discoidin domain receptor 1 drives progression of TP53-Mutant cancers
Source: J Biol Chem. 2025 Jul 24;301(9):110515. doi: 10.1016/j.jbc.2025.110515 (PMC12390941; doi:10.1016/j.jbc.2025.110515)

# Supplementary Figure 2

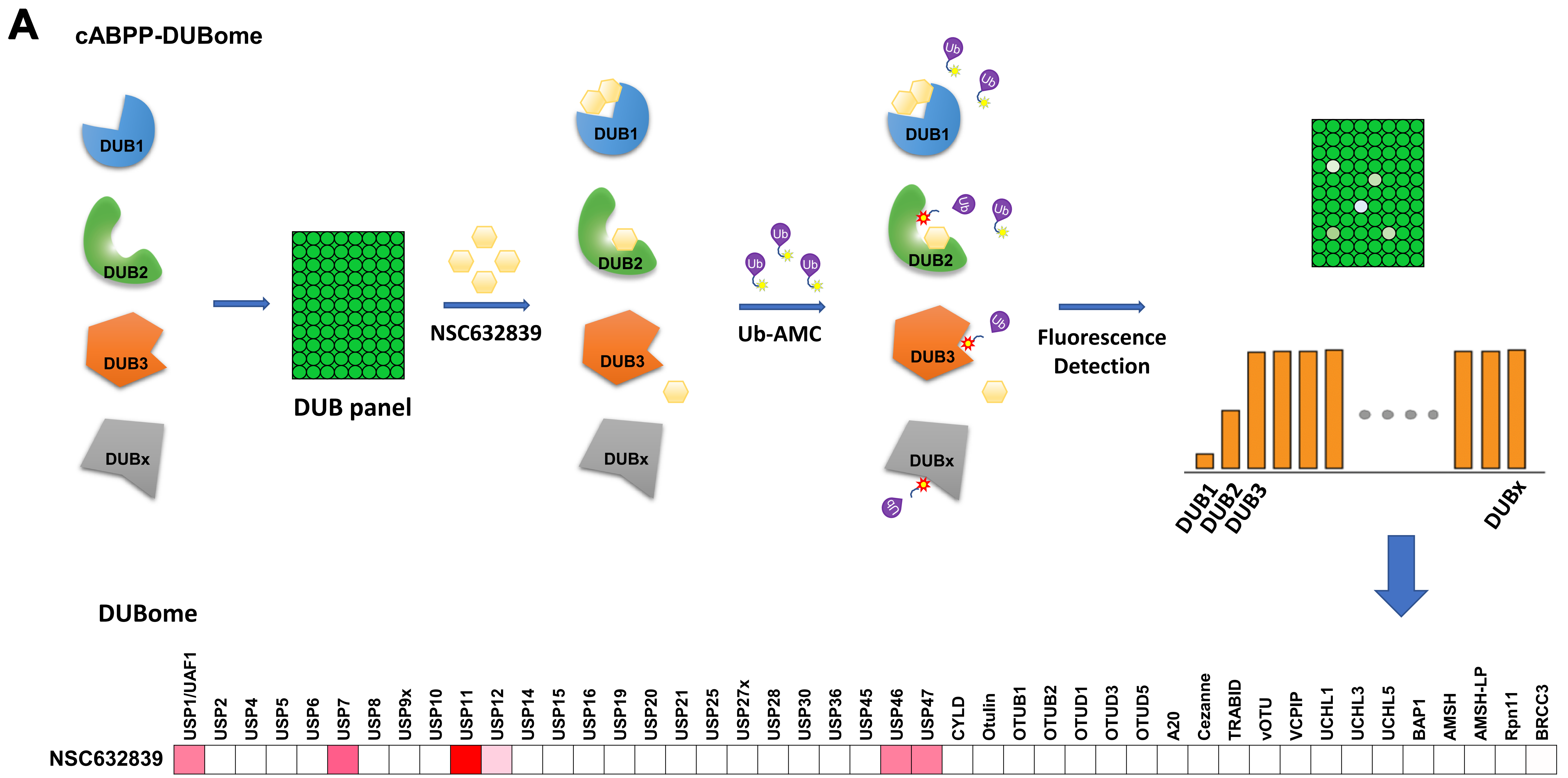


## B

LC-MS

| Identified Protein | Unique Peptides | Coverage (%) | Unique intensity |
|---|---|---|---|
| **USP7** | 4 | 2.99 | 1.24E+07 |
| USP10 | 3 | 3.88 | 2.98E+07 |
| TRIM25 | 2 | 3.17 | 1.20E+07 |
| TRIM47 | 2 | 3.29 | 3719728 |

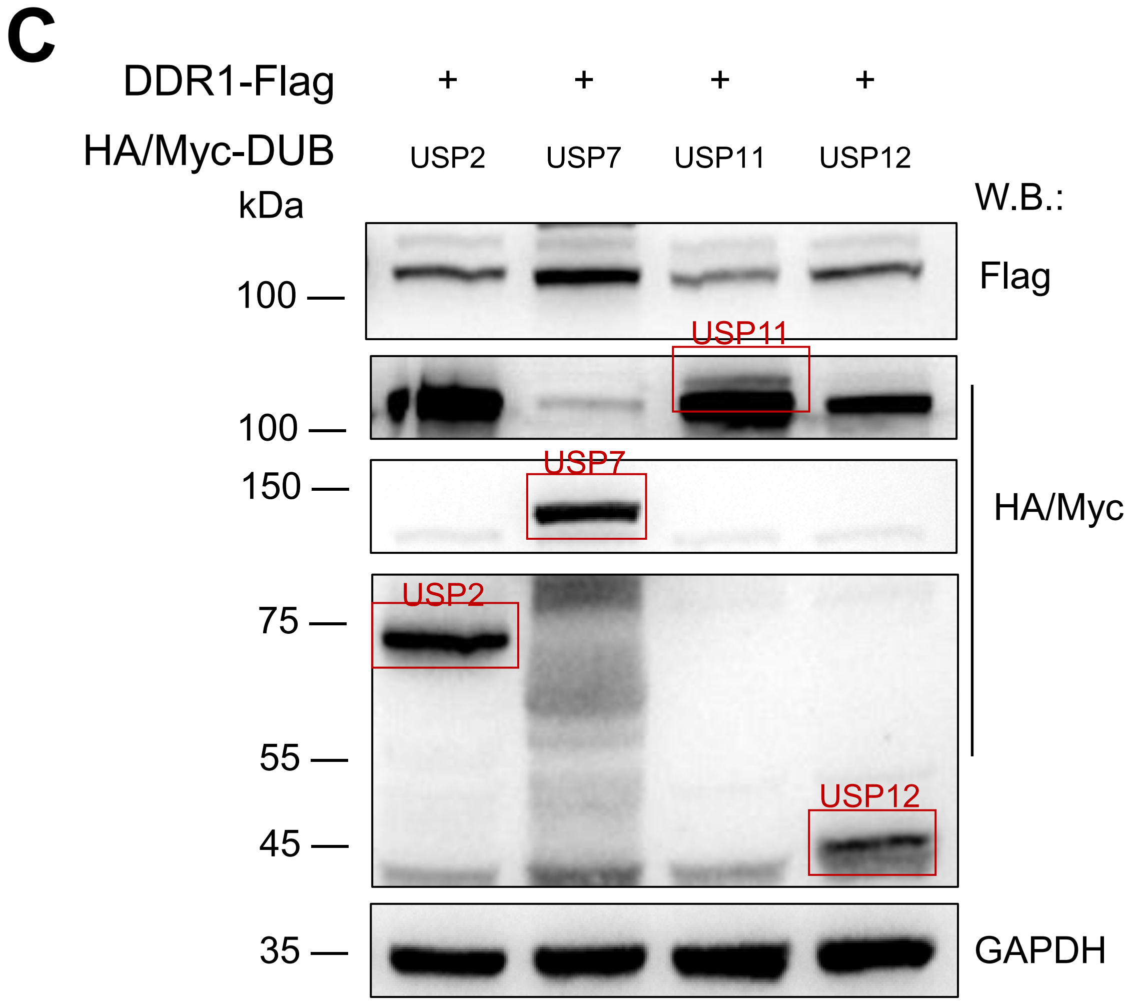

Supplement: Supplementary Figure 2 [file mmc3.pdf]
